# Unconventional height functions in simultaneous Diophantine approximation

**DOI:** 10.1007/s00605-016-0983-0

**Published:** 2016-10-18

**Authors:** Lior Fishman, David Simmons

**Affiliations:** 1 Department of Mathematics, University of North Texas, 1155 Union Circle #311430, Denton, TX 76203-5017 USA; 2 Department of Mathematics, University of York, Heslington, York YO10 5DD UK

**Keywords:** Diophantine approximation, Height functions, Dirichlet’s theorem, Continued fractions, Hardy *L*-functions, Primary 11J04, 11J13, 11J70, 34C11, 34C12

## Abstract

Simultaneous Diophantine approximation is concerned with the approximation of a point $\mathbf x\in \mathbb R^d$ by points $\mathbf r\in \mathbb Q^d$, with a view towards jointly minimizing the quantities $\Vert \mathbf x - \mathbf r\Vert$ and $H(\mathbf r)$. Here $H(\mathbf r)$ is the so-called “standard height” of the rational point $\mathbf r$. In this paper the authors ask: What changes if we replace the standard height function by a different one? As it turns out, this change leads to dramatic differences from the classical theory and requires the development of new methods. We discuss three examples of nonstandard height functions, computing their exponents of irrationality as well as giving more precise results. A list of open questions is also given.

Fix $d\ge 1$, and for each function $\Theta :\mathbb N^d\rightarrow \mathbb N$ let $H_{\Theta }:\mathbb Q^d\rightarrow \mathbb N$ be defined by the formula

$$\begin{aligned} H_{\Theta }\left( \frac{p_1}{q_1},\ldots ,\frac{p_d}{q_d}\right) = \Theta (q_1,\ldots ,q_d). \end{aligned}$$

Here we assume that $p_1/q_1,\ldots ,p_d/q_d\in \mathbb Q$ are given in reduced form. The function $H_{\Theta }$ will be called a *height function* on $\mathbb Q^d$.

Classical simultaneous Diophantine approximation is concerned with the *standard height function* $H_{\mathtt{lcm}}$, where $\mathtt{lcm}:\mathbb N^d\rightarrow \mathbb N$ is the least common multiple function. Historically, this height function and its variations and generalizations (see e.g. [6, §VIII.5-6]) have played a major role in modern mathematics, not only in Diophantine approximation but also in the theories of projective varieties and elliptic curves.^1^ The standard height function has been treated as the natural choice for a height function on $\mathbb Q^d$, to the point where no other choices were even considered. One reason for the historical emphasis on the standard height function is its connection to the lattice $\mathbb Z^d$; specifically; given $\mathbf r\in \mathbb Q^d$, $H_{\mathtt{lcm}}(\mathbf r)$ is the smallest number *q* such that $\mathbf r= \mathbf p/q$ for some $\mathbf p\in \mathbb Z^d$. This way of interpreting $H_{\mathtt{lcm}}$ lends itself more easily to generalizations to projective varieties and algebraic number fields; cf. [6, Remark VIII.5.5]. The connection to lattices also induces a connection between the Diophantine approximation based on this height function and the dynamics of the homogeneous space ${{\mathrm{SL}}}_{d + 1}(\mathbb R)/{{\mathrm{SL}}}_{d + 1}(\mathbb Z)$; cf. [5, Theorem 8.5].

The aim of this paper is to broaden the viewpoint of simultaneous Diophantine approximation by considering alternative height functions. Specifically, we will consider the height functions $H_{\mathtt{max}}$, $H_{\mathtt{min}}$, and $H_{\mathtt{prod}}$ defined by the maximum, minimum, and product functions $\mathtt{max},\mathtt{min},\mathtt{prod}:\mathbb N^d\rightarrow \mathbb N$.^2^ Although these height functions are not as related to the lattice $\mathbb Z^d$ (but see the Remark after Theorem 1.2 for a relation between the height functions $H_{\mathtt{prod}}$ and $H_{\mathtt{lcm}}$ based on the Segre embedding), in a certain sense they are more natural than $H_{\mathtt{lcm}}$, since the functions $\mathtt{max}$, $\mathtt{min}$, and $\mathtt{prod}$ are monotonic whereas $\mathtt{lcm}$ is not. Thus the study of these alternative height functions will be based not as much on the study of lattices, but will take a more “component-wise” approach.

The authors devote a section to analyzing a certain class of functions, the class of *recursively integrable functions* (denoted $\mathcal R$), which is used in the proof of one of the main theorems. The class $\mathcal R$ is contained in the class of integrable functions, and is similar to it in some ways. However, unlike the class of integrable functions, the class $\mathcal R$ is not closed under either addition or scalar multiplication. Nevertheless, there are many functions $f_2$ with the property that for every $f_1\in \mathcal R$, we have $f_1 + f_2\in \mathcal R$.

**Convention 1.** For $\alpha \ge 0$, we let $\psi _\alpha (q) = q^{-\alpha }$.

**Convention 2.** Given $\Theta :\mathbb N^d\rightarrow \mathbb N$ and $(q_i)_{i = 1}^d \in \mathbb N^d$, we will write

$$\begin{aligned} \Theta _{i = 1}^d q_i := \Theta (q_1,\ldots ,q_d). \end{aligned}$$

**Convention 3.** The symbols $\lesssim$, $\gtrsim$, and $\asymp$ will denote multiplicative asymptotics. For example, $A\lesssim _K B$ means that there exists a constant $C > 0$ (the *implied constant*), depending only on *K*, such that $A\le CB$.

**Convention 4.** In this paper “increasing” means “nondecreasing” and “decreasing” means “nonincreasing”, unless the word “strictly” is added.

**Convention 5.** The symbol $\triangleleft$ will be used to indicate the end of a nested proof.

## Main results

Throughout, $d\ge 1$ is fixed, and $\Vert \cdot \Vert$ denotes the max norm on $\mathbb R^d$. Note that if $d = 1$, then $H_{\mathtt{lcm}}= H_{\mathtt{max}}= H_{\mathtt{min}}= H_{\mathtt{prod}}= H_0$, where $H_0(p/q) = q$.

We begin by recalling Dirichlet’s theorem:

### Theorem

(Dirichlet’s Approximation Theorem) For each $\mathbf x\in \mathbb R^d$, and for any $Q \in \mathbb N$, there exists $\mathbf p/q\in \mathbb Q^d$ with $1\le q \le Q^d$ such that

$$\begin{aligned} \left\| \mathbf x- \frac{\mathbf p}{q} \right\| < \frac{1}{qQ}\cdot \end{aligned}$$

### Corollary

(Dirichlet’s Corollary) For every $\mathbf x\in \mathbb R^d{\setminus }\mathbb Q^d$,

$$\begin{aligned} \left\| \mathbf x- \frac{\mathbf p}{q}\right\| < \frac{1}{q^{1 + 1/d}} \text { for infinitely many } \frac{\mathbf p}{q} \in \mathbb Q^d. \end{aligned}$$

Equivalently^3^,

1.1 $$\begin{aligned} \Vert \mathbf x- \mathbf r_n \Vert < \psi _{1 + 1/d}\circ H_{\mathtt{lcm}}(\mathbf r_n) \text { for some sequence } \mathbb Q^d\ni \mathbf r_n\rightarrow \mathbf x. \end{aligned}$$

In what follows, we consider analogues of Dirichlet’s Corollary when $H_{\mathtt{lcm}}$ is replaced by one of the three height functions $H_{\mathtt{max}}$, $H_{\mathtt{min}}$, and $H_{\mathtt{prod}}$.

### Exponents of irrationality

Before getting down to the details of our main theorems, we first consider “coarse” analogues of Dirichlet’s Corollary. Specifically, we determine what the appropriate analogue of the exponent $1 + 1/d$ which appears in the formula (1.1) should be for our nonstandard height functions. More precisely:

#### Definition

Given a height function $H:\mathbb Q^d\rightarrow \mathbb N$ and a point $\mathbf x\in \mathbb R^d{\setminus }\mathbb Q^d$, the *exponent of irrationality* of $\mathbf x$ is

$$\begin{aligned} \omega _H(\mathbf x) = \liminf _{\begin{array}{c} \mathbf r\in \mathbb Q^d \\ \mathbf r\rightarrow \mathbf x \end{array}} \frac{-\log \Vert \mathbf x- \mathbf r\Vert }{\log H(\mathbf r)} = \lim _{\varepsilon \rightarrow 0} \inf _{\begin{array}{c} \mathbf r\in \mathbb Q^d \\ \Vert \mathbf x- \mathbf r\Vert \le \varepsilon \end{array}} \frac{-\log \Vert \mathbf x- \mathbf r\Vert }{\log H(\mathbf r)}\cdot \end{aligned}$$

Equivalently, $\omega _H(\mathbf x)$ is the supremum of all $\alpha \ge 0$ such that

$$\begin{aligned} \left\| \mathbf x- \mathbf r_n\right\| < \psi _\alpha \circ H(\mathbf r_n) \text { for some sequence } \mathbb Q^d \ni \mathbf r_n \rightarrow \mathbf x. \end{aligned}$$

The *exponent of irrationality* of the height function *H* is the number

$$\begin{aligned} \omega _d(H) = \inf _{\mathbf x\in \mathbb R^d{\setminus }\mathbb Q^d} \omega _H(\mathbf x). \end{aligned}$$

We observe that Dirichlet’s Corollary implies that $\omega _d(H_{\mathtt{lcm}}) \ge 1 + 1/d$. In fact, the reverse inequality is true (and well-known):

$$\begin{aligned} \omega _d(H_{\mathtt{lcm}}) = 1 + 1/d. \end{aligned}$$

This means that $1 + 1/d$ is the “best exponent” that can be put into formula (1.1).

We are now ready to state the following theorem regarding exponents of irrationality:

#### Theorem 1.1

(Exponents of irrationality of $H_{\mathtt{max}}$, $H_{\mathtt{min}}$, and $H_{\mathtt{prod}}$)

1.2 $$\begin{aligned}&\displaystyle \omega _d(H_{\mathtt{max}}) = \frac{d}{(d - 1)^{(d - 1)/d}} \text {if}\,\, d\ge 2\end{aligned}$$

1.3 $$\begin{aligned}&\displaystyle \omega _d(H_{\mathtt{min}}) = 2\end{aligned}$$

1.4 $$\begin{aligned}&\displaystyle \omega _d(H_{\mathtt{prod}}) = \frac{2}{d}\cdot \end{aligned}$$

#### Remark

The inequalities $\mathtt{min}\le \mathtt{prod}^{1/d}\le \mathtt{max}\le \mathtt{lcm}\le \mathtt{prod}$ automatically imply that

$$\begin{aligned} \omega _d(H_{\mathtt{prod}}) \le \omega _d(H_{\mathtt{lcm}}) \le \omega _d(H_{\mathtt{max}}) \le d \phantom {\cdot } \omega _d(H_{\mathtt{prod}}) \le \omega _d(H_{\mathtt{min}}). \end{aligned}$$

Theorem 1.1 shows that when $d\ge 3$, all inequalities are strict except the last. (When $d = 2$, the third inequality is also not strict.) It is also interesting to note that $\lim _{d\rightarrow \infty }\omega _d(H_{\mathtt{max}}) = 1 = \lim _{d\rightarrow \infty }\omega _d(H_{\mathtt{lcm}})$, so the second inequality is asymptotically an equality.

### More precise results

We now prepare to state our main theorems. These theorems will answer the question of what the appropriate analogue of the function $\psi _{1 + 1/d}$ should be for our nonstandard height functions. More precisely:

#### Definition

Given a height function $H:\mathbb Q^d\rightarrow \mathbb N$, a function $\psi :\mathbb N\rightarrow (0,\infty )$, and a point $\mathbf x\in \mathbb R^d$, let

1.5 $$\begin{aligned} C_{H,\psi }(\mathbf x) = \liminf _{\begin{array}{c} \mathbf r\in \mathbb Q^d \\ \mathbf r\rightarrow \mathbf x \end{array}} \frac{\Vert \mathbf x- \mathbf r\Vert }{\psi \circ H(\mathbf r)}\cdot \end{aligned}$$

Equivalently, $C_{H,\psi }(\mathbf x)$ is the infimum of all $C\ge 0$ such that

$$\begin{aligned} \Vert \mathbf x- \mathbf r_n\Vert < C \psi \circ H(\mathbf r_n) \quad \text {for some sequence} \mathbb Q^d\ni \mathbf r_n\rightarrow \mathbf x. \end{aligned}$$

A function $\psi$ will be called *Dirichlet* on $\mathbb R^d$ with respect to the height function *H* if $C_{H,\psi }(\mathbf x) < \infty$ for all $\mathbf x\in \mathbb R^d{\setminus }\mathbb Q^d$, *uniformly Dirichlet* if $\sup _{\mathbb R^d{\setminus }\mathbb Q^d} C_{H,\psi } < \infty$, and *optimally Dirichlet* if $\psi$ is Dirichlet and $C_{H,\psi }(\mathbf x) > 0$ for at least one $\mathbf x\in \mathbb R^d{\setminus }\mathbb Q^d$. (This terminology originally appeared in [2].)

We observe that Dirichlet’s Corollary implies that the function $\psi _{1 + 1/d}$ is uniformly Dirichlet on $\mathbb R^d$ with respect to the height function $H_{\mathtt{lcm}}$, and in fact that

$$\begin{aligned} C_{H_{\mathtt{lcm}},\psi _{1 + 1/d}}(\mathbf x) \le 1 \;\;\forall \mathbf x\in \mathbb R^d{\setminus }\mathbb Q^d. \end{aligned}$$

In fact, the function $\psi _{1 + 1/d}$ is optimally Dirichlet on $\mathbb R^d$ with respect to the height function $H_{\mathtt{lcm}}$, due to the existence of so-called *badly approximable vectors*, i.e. vectors $\mathbf x\in \mathbb R^d{\setminus }\mathbb Q^d$ for which $C_{H_{\mathtt{lcm}},\psi _{1 + 1/d}}(\mathbf x) > 0$. Roughly, the statement that $\psi _{1 + 1/d}$ is optimally Dirichlet should be interpreted as meaning that in formula (1.1), the function $\psi _{1 + 1/d}$ cannot be improved by more than a multiplicative constant. This interpretation was made rigorous in [2, Theorem 2.6 and Proposition 2.7].

#### Example

The function $\psi _2(q) = q^{-2}$ is uniformly and optimally Dirichlet on $\mathbb R$ with respect to the height function $H_0$. This fact may be equivalently expressed as follows:

(i) ($\psi _2$ is uniformly Dirichlet) There exists $C > 0$ such that for all $x\in \mathbb R$, there exist infinitely many $p/q\in \mathbb Q$ such that $|x - p/q| \le C q^{-2}$.
(ii) (Optimality) There exist $x\in \mathbb R$ and $\varepsilon > 0$ such that $|x - p/q| \ge \varepsilon q^{-2}$ for all but finitely many $p/q\in \mathbb Q$.

#### Remark

We will sometimes deal with functions $\psi$ which are not defined for all natural numbers, but only for sufficiently large numbers. In this case, the formula (1.5) may be interpreted as referring to an arbitrary extension of $\psi$ to $\mathbb N$; it is clear that the precise nature of the extension does not matter.

Given a height function $H \in \{H_{\mathtt{max}},H_{\mathtt{min}},H_{\mathtt{prod}}\}$ and $d\ge 1$, we may now ask the following questions:

1. Is there an optimally Dirichlet function on $\mathbb R^d$ with respect to *H*?
2. If so, what is it?^4^
3. If not, can one give a criterion for determining whether or not a given function is Dirichlet?

It turns out that to answer these questions, we must consider two cases. The first case is when either $H \in \{H_{\mathtt{min}},H_{\mathtt{prod}}\}$ or $d \le 2$. In this case, the situation is similar to the situation for the height function $H_{\mathtt{lcm}}$: there is a uniformly and optimally Dirichlet function, and it comes from the class of power law functions $(\psi _\alpha )_{\alpha \ge 0}$. Precisely:

#### Theorem 1.2

Fix $\Theta \in \{\mathtt{max},\mathtt{min},\mathtt{prod}\}$, and if $\Theta = \mathtt{max}$ assume that $d \le 2$. Then the function

$$\begin{aligned} \psi _{\omega _d(H_{\Theta })}(q) = {\left\{ \begin{array}{ll} q^{-2} &{} \Theta = \mathtt{max},\mathtt{min}\\ q^{-2/d} &{} \Theta = \mathtt{prod}\end{array}\right. } \end{aligned}$$

is uniformly and optimally Dirichlet on $\mathbb R^d$ with respect to the height function $H_{\Theta }$.

#### Remark

The case $\Theta = \mathtt{prod}$ of Theorem 1.2 can be reformulated as a theorem about *intrinsic* Diophantine approximation (see e.g. [1]) using the *standard* height function $H_{\mathtt{lcm}}$ on the variety $M_d = \Phi _d(\mathbb R^d) \subset \mathbb R^{2^d - 1}$, where

$$\begin{aligned} \Phi _d(x_1,\ldots ,x_d) = \left( {\prod }_{i\in S} x_i\right) _{{\emptyset }\ne S \subset \{1,\ldots ,d\}} \end{aligned}$$

is (the affinization of) the Segre embedding. This is because for every rational $\mathbf r\in \mathbb Q^d$, we have $H_{\mathtt{prod}}(\mathbf r) = H_{\mathtt{lcm}}\circ \Phi _d(\mathbf r)$. In the terminology of [1], the reformulated theorem states that the function $\psi (q) = q^{-2/d}$ is an optimal Dirichlet function for the Diophantine triple $(M_d,\mathbb Q^{2^d - 1}\cap M_d,H_{\mathtt{lcm}})$. (It is uniformly Dirichlet on compact subsets of this triple.) The special case $d = 2$ follows from [1, Theorems 4.5 and 5.1] using the fact that $M_2$ is a quadric hypersurface; cf. [1, Remark 8.1].

In the second case, namely when $H = H_{\mathtt{max}}$ and $d \ge 3$, the situation is much different. Specifically, when $d\ge 3$ the height function $H_{\mathtt{max}}$ has the following unexpected property: It possesses no “reasonable” optimally Dirichlet function. To state this precisely, we need to define the class of functions that we consider to be reasonable. A *Hardy L-function* is a function which can be expressed using only the elementary arithmetic operations $+,-,\times ,\div$, exponents, logarithms, and real-valued constants, and which is well-defined on some interval of the form $(t_0,\infty )$.^5^ For example, for any $C,\alpha \ge 0$ the function

$$\begin{aligned} \psi (q) = q^{-\alpha + C/\log ^2\log (q)} \end{aligned}$$

is a Hardy *L*-function. We have the following:

#### Theorem 1.3

Suppose $d\ge 3$. Then no Hardy *L*-function is optimally Dirichlet on $\mathbb R^d$ with respect to the height function $H_{\mathtt{max}}$.

#### Remark

The class of Hardy *L*-functions includes almost all functions that one naturally encounters in dealing with “analysis at infinity”, except for those with oscillatory behavior.

This answers question 1 above, so we would like next to answer question 3. Namely, given $d\ge 3$ and a Hardy *L*-function $\psi$, how does one determine whether or not $\psi$ is Dirichlet on $\mathbb R^d$ with respect to $H_{\mathtt{max}}$? Our final theorem (Theorem 1.4) will be a complete answer to this question. However, since it is complicated to state, we approach this theorem by degrees. As a first approximation we give the following corollary, which considers the case of a single error term added to the function $\psi _{\omega _d(H_{\mathtt{max}})}$:

#### Corollary

(of Theorem 1.4) Suppose $d\ge 3$. For each $C > 0$ let

1.6 $$\begin{aligned} \psi (q) = q^{-\omega _d(H_{\mathtt{max}}) + C/\log ^2\log (q)}. \end{aligned}$$

Then $\psi$ is (non-optimally) Dirichlet on $\mathbb R^d$ with respect to $H_{\mathtt{max}}$ if and only if

1.7 $$\begin{aligned} C > \frac{d\gamma _d \log ^2(\gamma _d)}{8}, \end{aligned}$$

where $$\gamma _d = (d - 1)^{1/d} > 1$$.

In particular, letting $C = 0$, we see that the function $\psi _{\omega _d(H_{\mathtt{max}})}$ is not Dirichlet on $\mathbb R^d$ with respect to $H_{\mathtt{max}}$.

This corollary now provides us with motivation to state our final theorem. Let $\psi$ be the function defined by (1.6) when $C = d\gamma _d\log ^2(\gamma _d)/8$. We know that $\psi$ is not Dirichlet (on $\mathbb R^d$ with respect to $H_{\mathtt{max}}$), but that for any function of the form $\phi _\varepsilon (q) = q^{\varepsilon /\log ^2\log (q)}$, the product $\phi _\varepsilon \psi$ is Dirichlet. This suggests that there is a function $\phi$ which grows more slowly than any $\phi _\varepsilon$ such that the product $\phi \psi$ is still Dirichlet. What function can we multiply by? As it turns out, if

$$\begin{aligned} \phi (q) = q^{C/[\log ^2\log (q)\log ^2\log \log (q)]}, \end{aligned}$$

then $\phi \psi$ is Dirichlet if and only if (1.7) holds. At this point it is clear that this line of questioning can be pursued *ad infinitum*, leading to the following:

#### Theorem 1.4

Suppose that $d\ge 3$. Then for each $N\ge 1$ and $C \ge 0$, the function

$$\begin{aligned} \psi _{N,C}(q)= & {} q\wedge \left( -\omega _d(H_{\mathtt{max}}) + \frac{d\gamma _d\log ^2(\gamma _d)}{8} \left[ \sum _{n = 2}^N \prod _{i = 2}^n \left( \frac{1}{\log ^{(i)}(q)}\right) ^2 \right. \right. \\&\left. \left. \quad +\,C\prod _{i = 2}^{N + 1}\left( \frac{1}{\log ^{(i)}(q)}\right) ^2 \right] \right) \end{aligned}$$

is (non-optimally) Dirichlet on $\mathbb R^d$ with respect to $H_{\mathtt{max}}$ if and only if $C > 1$. Here $\gamma _d = (d - 1)^{1/d}$ as before, and $\log ^{(i)}$ denotes the *i*th iterate of the logarithm function. If $N = 1$, then the first summation is equal to 0 by convention.

The earlier corollary is precisely the special case $N = 1$ of Theorem 1.4.

#### Remark

It may not be entirely obvious that Theorem 1.4 is a complete answer to question 3 in the case of Hardy *L*-functions. Nevertheless, it is. Precisely: If $\psi$ is a Hardy *L*-function, then there exist $N\ge 1$ and $C\ge 0$ such that comparing $\psi$ with $\psi _{N,C}$ together with Theorem 1.4 allow one to determine whether or not $\psi$ is Dirichlet on $\mathbb R^d$ with respect to $H_{\mathtt{max}}$. For a proof of this, see Proposition 5.7.

For a version of Theorem 1.4 which goes slightly beyond Hardy *L*-functions, allowing $\psi$ to be a member of any Hardy field which contains the exponential and logarithm functions and is closed under composition, see Proposition 6.1.

### Techniques

The main technique of this paper is to generalize the correspondence between the continued fraction expansion of an irrational number and its Diophantine properties into higher dimensions. This is done by introducing the notion of a *data progression* corresponding to an irrational vector $\mathbf x$, which is a mathematical object that encodes information about the continued fraction expansions of all of the coordinates of $\mathbf x$. The Diophantine properties of $\mathbf x$ can then be related to properties of the corresponding data progression. For more details see 2.2.

In the case of the height function $H_{\mathtt{max}}$, this correspondence translates the question of which functions are Dirichlet into a question about whether data progressions satisfying certain inequalities exist. We answer this question by converting it into a question about whether certain differential equations have nonnegative solutions, leading to the concept of a *recursively integrable function*. This concept is interesting in its own right and we study it in detail in Sect. 5. In particular we give a complete characterization of which Hardy *L*-functions are recursively integrable (Proposition 5.7), which leads to the characterization of which functions are Dirichlet described above in Theorem 1.4.

### Summary of the paper

Section 2 contains preliminary results which are used in the proofs of our main theorems. In Sect. 3 we prove Theorem 1.2, as well as demonstrating formulas (1.3) and (1.4). Section 4 provides a motivation for the first formula of Theorem 1.1 without giving a rigorous proof. Section 5 is devoted to defining and analyzing the class of recursively integrable functions, a class which is used in the proof of Theorems 1.3 and 1.4. In Sect. 6 we prove Theorems 1.3 and 1.4, as well as demonstrating formula (1.2). Finally, a list of open questions is given in Sect. 7.

## Preliminaries

### Lemmas concerning continued fractions

We begin our preliminaries with two lemmas concerning continued fractions. The first states that for $x\in \mathbb R$, the convergents of the continued fraction expansion of *x* provide the best approximations to *x* as long as one is willing to accept a multiplicative error term.^6^ Hence the Diophantine properties of *x* essentially depend only on the denominators of these convergents. The second states that given any sequence of numbers increasing fast enough, there is a number *x* such that the denominators of the convergents of the continued fraction expansion of *x* are equal up to an asymptotic to the elements of this sequence. Together, the two lemmas say that from a (sufficiently coarse) Diophantine point of view, the properties of a number can be encoded by an increasing sequence of integers.

#### Remark

This section is mostly interesting if *x* is an irrational number. However, since the implied constants are supposed to be independent of *x*, the results are nontrivial even when *x* is rational.

#### Lemma 2.1

Fix $x\in \mathbb R$, and let $(p_n/q_n)_0^N$ be the convergents of the continued fraction expansion of *x* (so that $N = \infty$ if and only if $x\notin \mathbb Q$). Then for every $p/q\in \mathbb Q$, there exists $n\in \mathbb N$ so that

$$\begin{aligned} q\gtrsim q_n \text { and } \left| x - \frac{p}{q}\right| \gtrsim \left| x - \frac{p_n}{q_n}\right| \end{aligned}$$

(cf. Convention 3).

Before we begin the proof, we recall [4, Theorem 1] that if $(a_n)_0^N$ are the partial quotients of the continued fraction expansion of *x*, then

2.1 $$\begin{aligned} p_n&= a_n p_{n - 1} + p_{n - 2}\end{aligned}$$

2.2 $$\begin{aligned} q_n&= a_n q_{n - 1} + q_{n - 2} \end{aligned}$$

for all $n\ge 1$. Here we use the convention that $p_{-1} = 1$ and $q_{-1} = 0$. In particular, the sequence $(q_n)_0^N$ is strictly increasing and satisfies $q_n \asymp a_n q_{n - 1}$. We recall also [4, Theorems 9 and 13] that for all $0\le n < N$,

2.3 $$\begin{aligned} \left| x - \frac{p_n}{q_n}\right| \asymp \frac{1}{q_n q_{n + 1}}\cdot \end{aligned}$$

#### Proof

Consider the set $S = \{p'/q'\in \mathbb Q: q' \le q\}$, and let $p'/q'\in S$ be chosen to minimize $|x - p'/q'|$. Then $q'\le q$ and $|x - p'/q'| \le |x - p/q|$, so we may without loss of generality assume that $p/q = p'/q'$. In this case, *p* / *q* is a *best approximation of the first kind* in the sense of [4, p. 24]. By [4, Theorem 15], *p* / *q* is an *intermediate fraction* in the sense of [4, p. 14], i.e.

2.4 $$\begin{aligned} \frac{p}{q} = \frac{a p_{n - 1} + p_{n - 2}}{a q_{n - 1} + q_{n - 2}} \end{aligned}$$

for some $1\le n\le N$ and $1\le a\le a_n$. We consider two cases separately:

- Case 1: $a\ge a_n/2$. In this case,
  $$\begin{aligned} 2q \ge a_n q_{n - 1} + q_{n - 2} = q_n. \end{aligned}$$
  On the other hand, by [4, Theorem 17], $p_n/q_n$ is a best approximation of the second kind, and thus also a best approximation of the first kind. Since $q\le q_n$, this gives
  $$\begin{aligned} \left| x - \frac{p}{q}\right| > \left| x - \frac{p_n}{q_n}\right| , \end{aligned}$$
  completing the proof in this case.
- Case 2: $1\le a < a_n/2$. In this case, since *p* / *q* lies on the same side of *x* as $p_n/q_n$ (cf. [4, Theorem 4] and [4, Lemma on p.14]), we have
  $$\begin{aligned} \left| x - \frac{p}{q}\right|&\ge \left| \frac{p_n}{q_n} - \frac{p}{q}\right| \\&= \left| \frac{a_n p_{n - 1} + p_{n - 2}}{a_n q_{n - 1} + q_{n - 2}} - \frac{a p_{n - 1} + p_{n - 2}}{a q_{n - 1} + q_{n - 2}}\right| \\&= \frac{a_n - a}{[a_n q_{n - 1} + q_{n - 2}][a q_{n - 1} + q_{n - 2}]}&\text {(cf.} \ [4, \mathrm{Theorem\, 2}])\\&\ge \frac{a_n/2}{q_n^2} \asymp \frac{1}{q_{n - 1} q_n} \asymp \left| x - \frac{p_{n - 1}}{q_{n - 1}}\right| . \end{aligned}$$
  Since $q\ge q_{n - 1}$, this completes the proof in this case.$\square$

#### Lemma 2.2

Let $(\widetilde{q}_n)_0^N$ be a (finite or infinite) sequence satisfying $\widetilde{q}_{n + 1}\ge 2 \widetilde{q}_n$ and $\widetilde{q}_0 = 1$. Then there exists $x\in \mathbb R$ so that if $(p_n/q_n)_0^N$ are the convergents of the continued fraction expansion of *x*, then

2.5 $$\begin{aligned} \frac{1}{2}\widetilde{q}_n \le q_n \le \widetilde{q}_n \;\;\forall n\in \mathbb N. \end{aligned}$$

#### Proof

The proof will proceed by recursively defining a sequence of integers $(a_n)_1^N$ and then letting *x* be the unique number in (0, 1) whose partial quotients are given by $(a_n)_1^N$. Note that once this process is completed, for every $1\le M\le N$ the value of $q_M$ can be computed from (2.2) using only the data points $(a_n)_1^M$ together with the initial values $q_{-1} = 0$, $q_0 = 1$. Thus in our recursive step, once we have defined $(a_n)_1^M$, we may treat $(q_n)_1^M$ as also defined.

Fix $1\le M\le N$, and suppose that the values $(a_n)_1^{M - 1}$ have been fixed, and that the resulting values $(q_n)_1^{M - 1}$ all satisfy (2.5). In particular, when $n = M - 1$, (2.5) holds. (If $M = 1$, this is due to the assumption on $\widetilde{q}_0$ rather than to the induction hypothesis.) Let $a_M$ be the largest integer $a\ge 1$ such that $a q_{M - 1} + q_{M - 2} \le \widetilde{q}_M$. Such an integer exists because

$$\begin{aligned} \widetilde{q}_M \ge 2\widetilde{q}_{M - 1} \ge 2 q_{M - 1} \ge q_{M - 1} + q_{M - 2}. \end{aligned}$$

Let $q_M$ be given by (2.2). Then

$$\begin{aligned} q_M\le \widetilde{q}_M \le (a_M + 1) q_{M - 1} + q_{M - 2} \le 2 (a_M q_{M - 1} + q_{M - 2}) = 2 q_M, \end{aligned}$$

i.e. (2.5) holds when $n = M$. This completes the recursive step. $\square$

### Data progressions

Fix $d\ge 1$. In the previous section, we learned how the Diophantine properties of an irrational number *x* are encoded in the sequence of denominators of the convergents of the continued fraction expansion of *x*. Continuing with this theme, given an irrational point $\mathbf x\in \mathbb R^d{\setminus }\mathbb Q^d$ we would like to find a structure which encodes the Diophantine properties of $\mathbf x$. It turns out that the appropriate structure for this encoding is given by the following definition:

#### Definition 2.3

Let $\Delta = (A_k,i_k)_{k = 1}^\infty$ be a pair of sequences, so that $A_k\in \mathbb R$ and $i_k\in \{1,\ldots ,d\}$ for all $k\in \mathbb N$. Assume that $\{i_k : k\in \mathbb N\} = \{1,\ldots ,d\}$. For each $i = 1,\ldots ,d$ and *k* sufficiently large, let

$$\begin{aligned} \ell (i,k)&:= \max \{k' < k : i_{k'} = i\}\\ b_k^{(i)}&:= A_{\ell (i,k) + 1}. \end{aligned}$$

Equivalently, the sequence $(\Delta _k := (b_k^{(i)})_{i = 1}^d)_{k = 1}^\infty$ may be defined via the recursive formula

2.6 $$\begin{aligned} b_{k + 1}^{(i)} = {\left\{ \begin{array}{ll} A_{k + 1} &{}\quad \text {if } i = i_k\\ b_k^{(i)} &{}\quad \text {if } i \ne i_k \end{array}\right. }. \end{aligned}$$

We say that $\Delta$ is a *d*-*dimensional data progression* if the following hold:

(I) For all *k* sufficiently large,
  2.7 $$\begin{aligned} b_{k + 1}^{(i_k)} > b_k^{(i_k)}. \end{aligned}$$
(II) The sequence $(\max (\Delta _k))_{k = 1}^\infty$ is unbounded.

Given $\Xi :{[0,\infty )}^d \rightarrow {[0,\infty )}$ and $\Psi :{[0,\infty )}\rightarrow \mathbb R$ we write

$$\begin{aligned} C_{\Xi ,\Psi }(\Delta ) = \liminf _{k\rightarrow \infty } \left( \Psi \left( \Xi _{i = 1}^d b_k^{(i)}\right) - b_k^{(i_k)} - b_{k + 1}^{(i_k)}\right) . \end{aligned}$$

#### Remark

In the sequel, the notation introduced in this definition will be used without comment.

#### Remark

A pair of sequences $\Delta = (A_k,i_k)_{k = 1}^\infty$ is a one-dimensional data progression if and only if $i_k = 1$ for all *k*, and the sequence $(A_k)_{k = 1}^\infty$ is increasing and tends to infinity. The canonical example is the sequence $(q_k)_{k = 1}^\infty$ of denominators of convergents of an irrational number $x \in \mathbb R{\setminus }\mathbb Q$.

#### Lemma 2.4

Fix $\Theta :[1,\infty )^d\rightarrow [1,\infty )$ and $\psi :[1,\infty )\rightarrow (0,\infty )$. Let $\Xi = \log \Theta \exp$ and let $\Psi = -\log \psi \exp$. Suppose that $\Xi$ and $\Psi$ are uniformly continuous and coordinatewise increasing.

(i) For each $\mathbf x\in \mathbb R^d{\setminus }\mathbb Q^d$, there exists a *d*-dimensional data progression $\Delta$ such that
  2.8 $$\begin{aligned} C_{H_{\Theta },\psi }(\mathbf x) \lesssim \exp C_{\Xi ,\Psi }(\Delta ). \end{aligned}$$
(ii) Conversely, for each *d*-dimensional data progression $\Delta$, there exists $\mathbf x\in \mathbb R^d{\setminus }\mathbb Q^d$ such that
  2.9 $$\begin{aligned} C_{H_{\Theta },\psi }(\mathbf x) \gtrsim _{\psi ,\Theta } \exp C_{\Xi ,\Psi }(\Delta ). \end{aligned}$$

In particular

$$\begin{aligned} \sup _{\mathbb R^d{\setminus }\mathbb Q^d} C_{H_{\Theta },\psi } \asymp _{\psi ,\Theta } \exp \sup _\Delta C_{\Xi ,\Psi }(\Delta ), \end{aligned}$$

where the supremum is taken over all *d*-dimensional data progressions $\Delta$.

#### Remark

The maps $\mathbf x\mapsto \Delta$ and $\Delta \mapsto \mathbf x$ implicitly described in parts (i) and (ii) of Lemma 2.4, respectively, are in fact independent of $\Theta$ and $\psi$, as can be easily seen from the proof of Lemma 2.4. On an intuitive level these maps are “rough inverses” of each other, but we do not make this rigorous.

#### Remark 2.5

If $\Theta \in \{\mathtt{max},\mathtt{min},\mathtt{prod}\}$, then $\Xi \in \{\mathtt{max},\mathtt{min},\mathtt{sum}\}$ is uniformly continuous and coordinatewise increasing. If $\psi$ is a Hardy *L*-function whose decay is no faster than polynomial, then $\Psi$ is uniformly continuous and increasing (Lemma A.4). Thus for the situations considered in this paper, the hypotheses of Lemma 2.4 will be immediately satisfied.

#### Proof of (i)

Fix $\mathbf x\in \mathbb R^d{\setminus }\mathbb Q^d$, and for each $i = 1,\ldots ,d$, let $\big (p_n^{(i)}/q_n^{(i)}\big )_{n = 1}^{N_i}$ be the convergents of the continued fraction expansion of $x_i$. Here $N_i\in \mathbb N\cup \{\infty \}$, with $N_i = \infty$ for at least one *i*. Let $E_i = \{1,\ldots ,N_i - 1\}$ if $N_i \in \mathbb N$, and $E_i = \mathbb N$ if $N_i = \infty$. Let $E = \{(n,i) : i = 1,\ldots ,d, \; n\in E_i\}$, and define a map $f:E\rightarrow \mathbb N$ by letting $f(n,i) = q_n^{(i)}q_{n + 1}^{(i)}$. Let $\big ((m_k,i_k)\big )_{k = 1}^\infty$ be an indexing of *E* such that the map $k\mapsto f(m_k,i_k)$ is increasing. Then for each $k\in \mathbb N$, let

$$\begin{aligned} A_{k + 1} = \log (q_{m_k + 1}^{(i_k)}), \end{aligned}$$

and let $\Delta = (A_k,i_k)_{k = 1}^\infty$. Then $b_k^{(i_k)} = \log (q_{m_k}^{(i_k)})$ and $b_{k + 1}^{(i_k)} = \log (q_{m_k + 1}^{(i_k)})$. It follows immediately that $\Delta$ is a *d*-dimensional data progression. To demonstrate (2.8), let

$$\begin{aligned} L(i,k)&= \min \{k'\in \mathbb N: k' \ge k, i_{k'} = i\}\\ n(i,k)&= m_{L(i,k)} = m_{\ell (i,k)} + 1, \end{aligned}$$

so that

$$\begin{aligned} b_k^{(i)} = \log (q_{n(i,k)}^{(i)}). \end{aligned}$$

Now

$$\begin{aligned} \min _{i = 1}^d\left( q_{n(i,k)}^{(i)}q_{n(i,k) + 1}^{(i)}\right)&= \min _{i = 1}^d f(n(i,k),i)\\&= \min _{i = 1}^d f(m_{L(i,k)},i_{L(i,k)}) \\&= f(m_k,i_k) (since L(i_k,k) = k, \text {and} L(i,k) \ge k \text {for all} i\\&= q_{m_k}^{(i_k)} q_{m_k + 1}^{(i_k)}\\&= \exp (b_k^{(i_k)} + b_{k + 1}^{(i_k)}). \end{aligned}$$

Let $$\mathbf r_k = \Big (p_{n(i,k)}^{(i)} / q_{n(i,k)}^{(i)}\Big )_{i = 1}^d$$. Then

$$\begin{aligned} C_{H_{\Theta },\psi }(\mathbf x)&\le \liminf _{k\rightarrow \infty } \frac{\Vert \mathbf x- \mathbf r_k\Vert }{\psi \circ H_{\Theta }(\mathbf r_k)}\\&\asymp \liminf _{k\rightarrow \infty } \max _{i = 1}^d \frac{1}{q_{n(i,k)}^{(i)}q_{n(i,k) + 1}^{(i)}}\frac{1}{\psi \circ H_{\Theta }(\mathbf r_k)} (\mathrm{by} (2.3))\\&= \liminf _{k\rightarrow \infty } \frac{1}{\exp (b_k^{(i_k)} + b_{k + 1}^{(i_k)})}\frac{1}{\psi \left( \Theta _{i = 1}^d q_{n(i,k)}^{(i)}\right) } = \exp C_{\Xi ,\Psi }(\Delta ). &\end{aligned}$$

$$\square $$

#### Proof of (ii)

Let $\Delta = (A_k,i_k)_{k = 1}^\infty$ be a *d*-dimensional data progression. For each $i = 1,\ldots ,d$, define an increasing sequence $(k(i,n))_{n = 0}^{N_i}$ recursively: Let *k*(*i*, 0) be large enough so that $b_{k(i,0)}^{(i)}$ is defined. Now fix $n\ge 0$, and suppose that *k*(*i*, *n*) has been defined. Let $k(i,n + 1)$ be the smallest value of *k* such that

$$\begin{aligned} b_k^{(i)} \ge b_{k(i,n)}^{(i)} + \log (2) \end{aligned}$$

if such a value exists; otherwise let $N_i = n$. Then by Lemma 2.2, there exists $x_i\in \mathbb R$ satisfying

$$\begin{aligned} q_n^{(i)} \asymp \exp (b_{k(i,n)}^{(i)}) \;\;\forall 1\le n\le N_i, \end{aligned}$$

where $(p_n^{(i)}/q_n^{(i)})_{n = 1}^{N_i}$ are the convergents of the continued fraction expansion of $x_i$. By (II) of the definition of a data progression, we have $N_i = \infty$ for at least one *i* and thus $\mathbf x:= (x_1,\ldots ,x_d)\notin \mathbb Q^d$. We will demonstrate (2.9). Fix $\mathbf r\in \mathbb Q^d$. For each $i = 1,\ldots ,d$, by Lemma 2.1 there exists $n_i = n_i(\mathbf r)$ such that $H_0(r_i) \gtrsim q_{n_i}^{(i)}$ and $|x_i - r_i| \gtrsim |x - p_{n_i}^{(i)} / q_{n_i}^{(i)}|$. Let

$$\begin{aligned} k_i&= k_i(\mathbf r) = k(i,n_i(\mathbf r) + 1) - 1\\ k&= k(\mathbf r) = \min _{i = 1}^d k_i(\mathbf r), \end{aligned}$$

so that

$$\begin{aligned} b_{k(i,n_i)}^{(i)} \le b_{k_i}^{(i)} \le b_{k(i,n_i)}^{(i)} + \log (2). \end{aligned}$$

Here the understanding is that if $n_i = N_i$, then $k_i = \infty$ and $b_{k_i}^{(i)} = \lim _{k\rightarrow \infty } b_k^{(i)}$. Then

$$\begin{aligned} H_0(r_i) \gtrsim q_{n_i}^{(i)} \asymp \exp (b_{k(i,n_i)}^{(i)}) \asymp \exp (b_{k_i}^{(i)}) \ge \exp (b_k^{(i)}). \end{aligned}$$

Using the fact that $\Xi$ and $\Psi$ are uniformly continuous and coordinatewise increasing, we deduce that

$$\begin{aligned} \psi \circ H_{\Theta }(\mathbf r) = \psi \left( \Theta _{i = 1}^d H_0(r_i)\right) \lesssim _{\psi ,\Theta } \psi \left( \Theta _{i = 1}^d\exp (b_k^{(i)})\right) . \end{aligned}$$

On the other hand, for each *i* such that $k_i\ne \infty$ we have

$$\begin{aligned} |x_i - r_i| \gtrsim \left| x_i - \frac{p_{n_i}^{(i)}}{q_{n_i}^{(i)}}\right| \asymp \frac{1}{q_{n_i}^{(i)} q_{n_i + 1}^{(i)}} \asymp \frac{1}{\exp (b_{k(i,n_i)}^{(i)} + b_{k(i,n_i + 1)}^{(i)})} \asymp \frac{1}{\exp (b_{k_i}^{(i)} + b_{k_i + 1}^{(i)})}\cdot \end{aligned}$$

Since $i_{k_i} = i \;\;\forall i$, we have $k_{i_k} = k$. Thus

$$\begin{aligned} \Vert \mathbf x- \mathbf r\Vert \ge |x_{i_k} - r_{i_k}| \gtrsim \frac{1}{\exp (b_k^{(i_k)} + b_{k + 1}^{(i_k)})}\cdot \end{aligned}$$

Combining, we have

$$\begin{aligned} \frac{\Vert \mathbf x- \mathbf r\Vert }{\psi \circ H_{\Theta }(\mathbf r)} \gtrsim _{\psi ,\Theta } \frac{1}{\exp (b_k^{(i_k)} + b_{k + 1}^{(i_k)})}\frac{1}{\psi \left( \Theta _{i = 1}^d\exp (b_k^{(i)})\right) }\cdot \end{aligned}$$

Let $(\mathbf r_j)_1^\infty$ be a sequence in $\mathbb Q^d$ along which the liminf in (1.5) is achieved. Since $\Vert \mathbf x- \mathbf r_j\Vert \rightarrow 0$, it follows that for all $i = 1,\ldots ,d$, we have $n_i(\mathbf r_j)\rightarrow \infty$ and thus $k_i(\mathbf r_j)\rightarrow \infty$. So $k(\mathbf r_j)\rightarrow \infty$, and thus

$$\begin{aligned} C_{H_{\Theta },\psi }(\mathbf x)&=\lim _{j\rightarrow \infty } \frac{\Vert \mathbf x- \mathbf r_j\Vert }{\psi \circ H_\Theta (\mathbf r_j)}\\&\gtrsim _{\psi ,\Theta } \liminf _{k\rightarrow \infty } \frac{1}{\exp (b_k^{(i_k)} + b_{k + 1}^{(i_k)})}\frac{1}{\psi \left( \Theta _{i = 1}^d\exp (b_k^{(i)})\right) } = \exp C_{\Xi ,\Psi }(\Delta ). \end{aligned}$$

$$\square $$

## Proof of Theorem 1.2 and formulas (1.3), (1.4)

We begin by reformulating Theorem 1.2 using Theorem 1.1:

### Proposition 3.1

Fix $d\ge 1$ and $\Theta \in \{\mathtt{max},\mathtt{min},\mathtt{prod}\}$, and if $\Theta = \mathtt{max}$ assume that $d \le 2$. Let

3.1 $$\begin{aligned} \beta _d = {\left\{ \begin{array}{ll} 2 &{} \Theta = \mathtt{max},\mathtt{min}\\ 2/d &{} \Theta = \mathtt{prod}\end{array}\right. }\cdot \end{aligned}$$

Then $\psi _{\beta _d}$ is uniformly and optimally Dirichlet on $\mathbb R^d$ with respect to the height function $H_{\Theta }$.

Proving this reformulation is sufficient to prove Theorem 1.2. Indeed, Proposition 3.1 immediately implies that $\omega _d(H_{\Theta }) = \beta _d$; replacing $\beta _d$ by $\omega _d(H_{\Theta })$ in Proposition 3.1 yields Theorem 1.2.

Proposition 3.1 also implies (1.3) and (1.4), and the case $d = 2$ of (1.2).

### Remark

The case $d = 1$ of Proposition 3.1 merely states that $\psi _2$ is uniformly and optimally Dirichlet on $\mathbb R$ with respect to the standard height function $H_0$. Thus, in the proof we may assume $d\ge 2$.

### Proof of Uniform Dirichletness

By Lemma 2.4, it suffices to show that

$$\begin{aligned} \sup _\Delta C_{\Xi ,\Psi }(\Delta ) \le 1, \end{aligned}$$

where $\Xi = \log \Theta \exp$, $\Psi = -\log \psi _{\beta _d}\exp$, and the supremum is taken over *d*-dimensional data progressions $\Delta$. By contradiction suppose that $C_{\Xi ,\Psi }(\Delta ) > 1$ for some *d*-dimensional data progression $\Delta = (A_k,i_k)_{k = 1}^\infty$. Then for all *k* sufficiently large, we have

3.2 $$\begin{aligned} b_k^{(i_k)} + b_{k + 1}^{(i_k)} \le \beta _d\Xi _{i = 1}^d b_k^{(i)} - 1. \end{aligned}$$

Let ${{\mathrm{Var}}}(\Delta )$ and $\mathrm {Av}(\Delta )$ denote the variance and mean (average) of a *d*-tuple $\Delta$, respectively. Let $K = \{k\in \mathbb N: \max (\Delta _{k + 1}) > \max (\Delta _k)\}$. $\square$

### Claim 3.2

We have

3.3 $$\begin{aligned} {{\mathrm{Var}}}(\Delta _{k + 1})&\le {{\mathrm{Var}}}(\Delta _k) \;\;\forall k\in \mathbb N\end{aligned}$$

3.4 $$\begin{aligned} {{\mathrm{Var}}}(\Delta _{k + 1})&\le {{\mathrm{Var}}}(\Delta _k) - 1/\max (4,d) \;\;\forall k\in K. \end{aligned}$$

The proof is divided into two cases: either $\Theta \in \{\mathtt{min},\mathtt{prod}\}$, or $\Theta = \mathtt{max}$ and $d = 2$.

*Proof if* $\Theta \in \{\mathtt{min},\mathtt{prod}\}$. To begin with, we observe that

3.5 $$\begin{aligned} {{\mathrm{Var}}}(\Delta _{k + 1}) - {{\mathrm{Var}}}(\Delta _k)\le & {} \frac{1}{d}\sum _{i = 1}^d \left( b_{k + 1}^{(i)} - \mathrm {Av}(\Delta _k)\right) ^2 - {{\mathrm{Var}}}(\Delta _k)\nonumber \\= & {} \frac{1}{d}\left[ \left( b_{k + 1}^{(i_k)} - \mathrm {Av}(\Delta _k)\right) ^2 - \left( b_k^{(i_k)} - \mathrm {Av}(\Delta _k)\right) ^2\right] .\qquad \end{aligned}$$

Now by (3.2), we have

$$\begin{aligned} b_k^{(i_k)} + b_{k + 1}^{(i_k)} \le 2\mathrm {Av}(\Delta _k) - 1. \end{aligned}$$

(If $\Theta = \mathtt{prod}$, then this equation is simply a reformulation of (3.2); if $\Theta = \mathtt{min}$, it follows from the fact that $\min (\Delta _k) \le \mathrm {Av}(\Delta _k)$.) Rearranging gives

3.6 $$\begin{aligned} \mathrm {Av}(\Delta _k) \ge \frac{b_k^{(i_k)} + b_{k + 1}^{(i_k)}}{2} + \frac{1}{2}\cdot \end{aligned}$$

By (2.7), the above equation implies that

$$\begin{aligned} |b_{k + 1}^{(i_k)} - \mathrm {Av}(\Delta _k)| \le |b_k^{(i_k)} - \mathrm {Av}(\Delta _k)|. \end{aligned}$$

Combining with (3.5) completes the proof of (3.3). Now suppose that $k\in K$, and observe that $\mathrm {Av}(\Delta _k) \le \max (\Delta _k) < \max (\Delta _{k + 1}) = b_{k + 1}^{(i_k)}$. Combining with (3.6) yields

$$\begin{aligned} |b_{k + 1}^{(i_k)} - \mathrm {Av}(\Delta _k)| \le |b_k^{(i_k)} - \mathrm {Av}(\Delta _k)| - 1, \end{aligned}$$

and thus

$$\begin{aligned} |b_{k + 1}^{(i_k)} - \mathrm {Av}(\Delta _k)|^2 \le |b_k^{(i_k)} - \mathrm {Av}(\Delta _k)|^2 - 1. \end{aligned}$$

Combining with (3.5) gives (3.4).

*Proof if* $\Theta = \mathtt{max}$ and $d = 2$. In this case, (3.2) becomes

$$\begin{aligned} b_k^{(i_k)} + b_{k + 1}^{(i_k)} \le 2 \max (b_k^{(i_k)},b_k^{(j_k)}) - 1, \end{aligned}$$

where $j_k$ satisfies $\{i_k,j_k\} = \{1,2\}$. Combining with (2.7) gives $b_k^{(i_k)} < b_k^{(j_k)}$, and so rearranging gives

3.7 $$\begin{aligned} b_{k + 1}^{(j_k)} = b_k^{(j_k)} \ge \frac{b_k^{(i_k)} + b_{k + 1}^{(i_k)}}{2} + \frac{1}{2}. \end{aligned}$$

By (2.7), the above equation implies that

$$\begin{aligned} |b_{k + 1}^{(i_k)} - b_{k + 1}^{(j_k)}| = |b_{k + 1}^{(i_k)} - b_k^{(j_k)}| \le |b_k^{(i_k)} - b_k^{(j_k)}|, \end{aligned}$$

demonstrating (3.3). Now suppose that $k\in K$, and observe that $b_k^{(j_k)} = \max (\Delta _k) < \max (\Delta _{k + 1}) = b_{k + 1}^{(i_k)}$. Combining with (3.7) gives

$$\begin{aligned} |b_{k + 1}^{(i_k)} - b_{k + 1}^{(j_k)}| \le |b_k^{(i_k)} - b_k^{(j_k)}| - 1, \end{aligned}$$

and thus

$$\begin{aligned} |b_{k + 1}^{(i_k)} - b_{k + 1}^{(j_k)}|^2 \le |b_k^{(i_k)} - b_k^{(j_k)}|^2 - 1. \end{aligned}$$

Since ${{\mathrm{Var}}}(\Delta _k) = (1/4)|b_k^{(i_k)} - b_k^{(j_k)}|^2$, this equation is equivalent to (3.4). To complete the proof of Proposition 3.1, observe that *K* is infinite by (II) of Definition 2.3. Thus, it follows from Claim 3.2 that ${{\mathrm{Var}}}(\Delta _k) \rightarrow -\infty$. But this contradicts the fact that the variance of a data set is always nonnegative.

### Proof of Optimality

Let $x_1,\ldots ,x_d\in \mathbb R$ be badly approximable numbers, and let $\mathbf x= (x_1,\ldots ,x_d)$. We claim that $C_{H_{\Theta },\psi _{\beta _d}}(\mathbf x) > 0$, demonstrating the optimality of $\psi _{\beta _d}$. Indeed, for each $\mathbf r\in \mathbb Q^d$,

$$\begin{aligned} \Vert \mathbf x- \mathbf r\Vert = \max _{i = 1}^d \left| x_i - r_i\right| \gtrsim _\mathbf x\max _{i = 1}^d \frac{1}{H^2(r_i)} = \frac{1}{H_{\mathtt{min}}^2(\mathbf r)} \ge \frac{1}{H_{\mathtt{prod}}^{2/d}(\mathbf r)} \ge \frac{1}{H_{\mathtt{max}}^2(\mathbf r)}\cdot \end{aligned}$$

Thus $\Vert \mathbf x- \mathbf r\Vert \gtrsim _\mathbf x\psi _{\beta _d}\circ H_{\Theta }(\mathbf r)$, which implies the desired result. $\square$

## Interlude: Motivation for the value of $\omega _d(H_{\mathtt{max}})$

Before jumping into the proof of Theorems 1.3 and 1.4, in this section we try to motivate the formula (1.2). Our approach is as follows: The notion of a “data progression” is very broad, but it is natural to expect that “worst-case-scenario” data progressions will behave somewhat regularly. In fact, we will prove a rigorous version of this assertion in Sect. 6. But for now, let’s just see what happens if we restrict our attention to data progressions which behave regularly.

### Definition

A data progression $\Delta$ is *periodic* if the map $k\mapsto i_k$ is periodic of order *d*, and *geometric* if $A_k = \gamma ^k$ for some $\gamma > 1$. The number $\gamma$ is called the *mutliplier*.

### Remark

If a data progression is periodic, then the map $\{1,\ldots ,d\}\ni k\mapsto i_k$ must be a permutation.

### Remark

It is shown in Sect. 6 that to determine which functions $\psi$ are Dirichlet on $\mathbb R^d$ with respect to $H_{\mathtt{max}}$, it is sufficient to consider data progressions which are eventually periodic (Claim 6.6) and asymptotically geometric (Claim 6.5).

### Lemma 4.1

Let $\Delta$ be a periodic geometric *d*-dimensional data progression of multiplier $\gamma$. Fix $\alpha \ge 0$, and let $\Psi _\alpha (b) = \alpha b$. Then

$$\begin{aligned} C_{\mathtt{max},\Psi _\alpha }(\Delta ) = {\left\{ \begin{array}{ll} -\infty &{} \text {if } \gamma + \gamma ^{-(d - 1)} > \alpha \\ 0 &{} \text {if } \gamma + \gamma ^{-(d - 1)} = \alpha \\ \infty &{} \text {if } \gamma + \gamma ^{-(d - 1)} < \alpha \end{array}\right. }. \end{aligned}$$

### Proof

Since $\Delta$ is periodic, we have $\{b_k^{(i)} : i = 1,\ldots ,d\} = \{A_{k - j} : j = 0,\ldots ,d - 1\}$, $b_k^{(i_k)} = A_{k - d + 1}$, and $b_{k + 1}^{(i_k)} = A_{k + 1}$. Thus

$$\begin{aligned} C_{\mathtt{max},\Psi _\alpha }(\Delta )&= \liminf _{k\rightarrow \infty }\left( \alpha \max _{j = 0}^{d - 1} A_{k - j} - A_{k - d + 1} - A_{k + 1}\right) \\&= \liminf _{k\rightarrow \infty }\left( \alpha \gamma ^k - \gamma ^{k - d + 1} - \gamma ^{k + 1}\right) \\&= \liminf _{k\rightarrow \infty }\left( \alpha - \gamma ^{-(d - 1)} - \gamma \right) \gamma ^k. \end{aligned}$$

Since $\gamma ^k\rightarrow \infty$, this completes the proof. $\square$

Fix $\alpha \ge 0$. From Lemma 2.4, we know that $\psi _\alpha$ is Dirichlet on $\mathbb R^d$ with respect to $H_{\mathtt{max}}$ if and only if $C_{\mathtt{max},\Psi _\alpha }(\Delta ) < \infty$ for every *d*-dimensional data progression $\Delta$. Now comes the heuristic part: let’s figure out what happens if we consider only periodic geometric data progressions, rather than all data progressions.

### Proposition 4.2

The following are equivalent:

(A) $C_{\mathtt{max},\Psi _\alpha }(\Delta ) < \infty$ for every periodic geometric *d*-dimensional data progression $\Delta$.
(B) $$\alpha \le \alpha _d := d(d - 1)^{-(d - 1)/d}$$.

In light of Lemma 4.1, it suffices to prove the following:

### Lemma 4.3

The unique minimum of the function

$$\begin{aligned} f(\gamma ) = \gamma + \gamma ^{-(d - 1)} \end{aligned}$$

is attained at the value $\gamma _d = (d - 1)^{1/d}$, where it achieves the value $f(\gamma _d) = \alpha _d$.

The proof of this lemma is a calculus exercise which is left to the reader.

Note that $\gamma _d > 1$ if and only if $d\ge 3$. If $d = 2$, we still have $\sup _{\gamma > 1}f(\gamma ) = \alpha _d$ which is sufficient to deduce Proposition 4.2 from Lemma 4.1.

In the sequel, the following corollary will be useful:

### Corollary 4.4

The unique maximum of the function

$$\begin{aligned} f_d(\gamma ) = (\alpha _d - \gamma )\gamma ^{d - 1} \end{aligned}$$

is attained at the value $\gamma _d$, where it acheives the value $f_d(\gamma _d) = 1$.

### Proof

We have

$$\begin{aligned} \gamma + \gamma ^{-(d - 1)} \ge \alpha _d, \end{aligned}$$

with equality if and only if $\gamma = \gamma _d$; rearranging gives the desired result. $\square$

## The class of recursively integrable functions

In this section we introduce a class of functions to be used in the proof of Theorem 1.4, the class of *recursively integrable* functions.

### Definition 5.1

Fix $t_0 \ge 0$, and let $f:[t_0,\infty )\rightarrow {[0,\infty )}$ be a continuous function. We say that *f* is *recursively integrable* if for some $t_1 \ge t_0$ the differential equation

5.1 $$\begin{aligned} -g'(x) = g^2(x) + f(x) \end{aligned}$$

has a solution $g: [t_1,\infty )\rightarrow {[0,\infty )}$. The class of recursively integrable functions will be denoted $\mathcal R$. A solution *g* of (5.1) will be called a *recursive antiderivative* of *f* (regardless of its domain and range).

Note that if $f\in \mathcal R$, then *f* is integrable, since

$$\begin{aligned} \int _{t_1}^\infty f(x)\mathrm {d}x\le & {} \int _{t_1}^\infty [g^2(x) + f(x)]\mathrm {d}x \\= & {} -\int _{t_1}^\infty g'(x)\mathrm {d}x = g(t_1) - \lim _{t\rightarrow \infty } g(t) \le g(t_1) < \infty . \end{aligned}$$

Like the class of integrable functions, the class $\mathcal R$ is closed under $\le$:

### Lemma 5.2

If $0\le f_1 \le f_2$ and if $f_2\in \mathcal R$, then $f_1\in \mathcal R$.

### Proof

Let $g_2:[t_1,\infty )\rightarrow {[0,\infty )}$ be a recursive antiderivative of $f_2$. Let $g_1:[t_1,t_2)\rightarrow \mathbb R$ be a recursive antiderivative of $f_1$ satisfying $g_1(t_1) = g_2(t_1)$. Such a function $g_1$ exists by the fundamental theorem of ordinary differential equations; moreover, $t_2$ may be chosen so that either $t_2 = \infty$ or $\lim _{t\rightarrow t_2} g_1(t) = \pm \infty$. It is clear that $g_1 \ge g_2$. In particular $g_1 \ge 0$. On the other hand, $g_1$ is decreasing so $\lim _{t\rightarrow t_2} g_1(t)\ne +\infty$. Thus $t_2 = \infty$ and $g_1:[t_1,\infty )\rightarrow {[0,\infty )}$. $\square$

### Remark 5.3

Equivalently, Lemma 5.2 says that if the differential inequality

$$\begin{aligned} -g'(x) \ge g^2(x) + f(x) \end{aligned}$$

has a solution $g:[t_1,\infty )\rightarrow {[0,\infty )}$, then *f* is recursively integrable.

However, unlike the class of integrable functions, the class $\mathcal R$ is not closed under scalar multiplication, Indeed, we have:

### Lemma 5.4

Fix $C > 0$. The function $f(x) = C/x^2$ is recursively integrable if and only if $C\le 1/4$.

### Proof

Suppose that $C \le 1/4$. Then there exists $c > 0$ such that $C = c - c^2$. The function $g(x) = c/x$ is a recursive antiderivative of *f*, and thus $f\in \mathcal R$.

Conversely, suppose that $C > 1/4$, and by contradiction suppose that $g:[t_1,\infty )\rightarrow {[0,\infty )}$ is a recursive antiderivative of *f*. Letting $h(x) = xg(x)$, we have

$$\begin{aligned} \frac{h(x)}{x^2} - \frac{h'(x)}{x} = \frac{h^2(x)}{x^2} + \frac{C}{x^2}, \end{aligned}$$

or

$$\begin{aligned} -h'(x) = \frac{1}{x} \left[ h^2(x) - h(x) + C\right] . \end{aligned}$$

But since $C > 1/4$, there exists $\varepsilon > 0$ such that $y^2 - y + C \ge \varepsilon$ for all $y\in \mathbb R$. Thus

$$\begin{aligned} -h'(x) \ge \frac{\varepsilon }{x}\cdot \end{aligned}$$

It follows that $h(x)\rightarrow -\infty$ as $x\rightarrow \infty$, contradicting that $g:[t_0,\infty )\rightarrow {[0,\infty )}$. $\square$

If *f* is a function such that the limit $\lim _{x\rightarrow \infty } x^2 f(x)$ exists and is not equal to 1 / 4, then Lemmas 5.4 and 5.2 can be used to determine whether or not $f\in \mathcal R$. This leads to the question: what if $\lim _{x\rightarrow \infty } x^2 f(x) = 1/4$? The following lemma provides us with a tool to deal with such functions:

### Lemma 5.5

Let $f:[t_0,\infty )\rightarrow {[0,\infty )}$. Then $f\in \mathcal R$ if and only if $F\in \mathcal R$, where

$$\begin{aligned} F(x) := \frac{1}{x^2}\left[ \frac{1}{4} + f(\log (x))\right] . \end{aligned}$$

### Proof

For any function $g:[t_1,\infty )\rightarrow {[0,\infty )}$, let

5.2 $$\begin{aligned} G(x) := \frac{1}{x} \left[ \frac{1}{2} + g(\log (x))\right] . \end{aligned}$$

We havei.e. *G* is a recursive antiderivative of *F* if and only if *g* is a recursive antiderivative of *f*.

If $g:[t_1,\infty )\rightarrow {[0,\infty )}$ is a recursive antiderivative of *f*, let *G* be defined by (5.2). Since $G:[e^{t_1},\infty )\rightarrow {[0,\infty )}$, *F* is recursively integrable.

Conversely, suppose that $G:[t_1,\infty )\rightarrow {[0,\infty )}$ is a recursive antiderivative of *F*, with $t_1 > 0$. Let $g:[\log (t_1),\infty )\rightarrow [-1/2,\infty )$ be defined by (5.2); then *g* is a recursive antiderivative of *f*. To complete the proof we must show that *g* is nonnegative. But (5.1) together with the inequality $f\ge 0$ show that

5.3 $$\begin{aligned} -g'(x) \ge g^2(x) \ge 0. \end{aligned}$$

In particular *g* is decreasing. Since *g* is bounded from below, it follows that $\lim _{x\rightarrow \infty } g(x)$ exists. Applying (5.3) again, we see that this limit must equal 0. Since *g* is decreasing, this implies that $g(x)\ge 0$ for all *x*. $\square$

### Remark

An alternative proof of Lemma 5.4 may be given by applying Lemma 5.5 to the class of constant functions.

Applying Lemma 5.5 repeatedly to Lemma 5.4 yields the following:

### Corollary 5.6

For each $N\ge -1$ and $C\ge 0$, the function

$$\begin{aligned} f_{N,C}(x)&= \frac{1}{4}\sum _{n = 0}^N \prod _{i = 0}^n \left( \frac{1}{\log ^{(i)}(x)}\right) ^2 + C\prod _{i = 0}^{N + 1}\left( \frac{1}{\log ^{(i)}(x)}\right) ^2\\&= \frac{1}{x^2}\left[ \frac{1}{4} + \frac{1}{\log ^2(x)}\left[ \frac{1}{4} + \cdots + \left( \frac{1}{\log ^{(N)}(x)}\right) ^2\left[ \frac{1}{4} + C\left( \frac{1}{\log ^{(N + 1)}(x)}\right) ^2\right] \cdots \right] \right] \end{aligned}$$

is recursively integrable if and only if $C \le 1/4$. (If $N = -1$, then the first summation is equal to 0 by convention.)

### Remark

There is a resemblance between Corollary 5.6 and the following well-known theorem: For each $N\ge -1$ and $\alpha \ge 0$, the function

$$\begin{aligned} f(x)&= \left( \prod _{i = 0}^N \frac{1}{\log ^{(i)}(x)}\right) \left( \frac{1}{\log ^{(N + 1)}(x)}\right) ^\alpha \\&= \frac{1}{x\log (x)\cdots \log ^{(N)}(x) \left( \log ^{(N + 1)}(x)\right) ^\alpha } \end{aligned}$$

is integrable on an interval of the form $[t_0,\infty )$ if and only if $\alpha > 1$.

We next show that Corollary 5.6 can be used to determine whether or not $f\in \mathcal R$ whenever *f* is a Hardy *L*-function.

### Proposition 5.7

If *f* is a Hardy *L*-function, then there exist $N\in \mathbb N$ and $C \ge 0$ such that

5.4 $$\begin{aligned} f(x) \le f_{N,C}(x) \ \mathrm{for \ all}\ x\ \mathrm{sufficiently \ large \ if}\ C\le 1/4 \end{aligned}$$

and

5.5 $$\begin{aligned} f(x) \ge f_{N,C}(x) \ \mathrm{for \ all}\ x\ \mathrm{sufficiently \ large \ if}\ C > 1/4. \end{aligned}$$

We have $f\in \mathcal R$ or $f\notin \mathcal R$ according to whether the former or the latter holds.

The second assertion is of course a direct consequence of Corollary 5.6 and Lemma 5.2.

### Proof

Let *N* be the *order* of *f* as defined in [3, §4], and consider the function

$$\begin{aligned} g(x) = \prod _{i = 0}^N\left( \log ^{(i)}(x)\right) ^2 \left[ 4f(x) - \sum _{n = 0}^N \prod _{i = 0}^n \left( \frac{1}{\log ^{(i)}(x)}\right) ^2\right] . \end{aligned}$$

Note that for each $C \ge 0$, we have $f(x) \le f_{N,C}(x)$ if and only if $g(x) \le 4C(\log ^{(n + 1)}(x))^{-2}$. On the other hand, it is readily seen that *g* is a Hardy *L*-function of order $\le N$. So by [3, Theorem 3], there exists $\varepsilon > 0$ such that either

$$\begin{aligned} g(x) \le \left( \log ^{(N)}(x)\right) ^{-\varepsilon } \ \mathrm{for \ all}\ x \ \mathrm{sufficiently \ large}, \end{aligned}$$

or

$$\begin{aligned} g(x) \ge \varepsilon \ \mathrm{for \ all}\ x\ \mathrm{sufficiently \ large}. \end{aligned}$$

In the first case, we have $g(x) \le (\log ^{(n + 1)}(x))^{-2}$ for all *x* sufficiently large, so (5.4) holds with $C = 1/4$. In the second case, we have $g(x) \ge 2(\log ^{(n + 1)}(x))^{-2}$ for all *x* sufficiently large, so (5.5) holds with $C = 1/2$. $\square$

One more fact about transformations preserving recursive integrability will turn out to be useful:

### Lemma 5.8

Fix $\lambda > 0$. A function $f:[t_0,\infty )\rightarrow {[0,\infty )}$ is recursively integrable if and only if the function

$$\begin{aligned} f_\lambda (x) = \lambda ^2 f(\lambda x) \end{aligned}$$

is recursively integrable.

### Proof

If *g* is a recursive antiderivative of *f*, then $g_\lambda (x) = \lambda g(\lambda x)$ is a recursive antiderivative of $f_\lambda$. Since $f = (f_\lambda )_{1/\lambda }$, the backwards direction follows from the forwards direction. $\square$

We next discuss the robustness of the concept of recursive integrability. As we have seen, it is not preserved under scalar multiplication. In particular, the sum of two recursively integrable functions is not necessarily recursively integrable. However, there are certain functions which can be safely added to a recursively integrable function without affecting its recursive integrability.

In what follows, $\mathcal H$ denotes a Hardy field (cf. Appendix 1) which contains the exponential and logarithm functions and is closed under composition. For example, $\mathcal H$ can be (and must contain) the class of Hardy *L*-functions described in the introduction.

### Definition 5.9

A nonnegative function $f_2\in \mathcal H$ is *ignorable* if for every function $f_1\in \mathcal R\cap \mathcal H$, we have $f_1 + f_2\in \mathcal R$.

Note that the sum of any two ignorable functions is ignorable. Moreover, if $f_2$ is ignorable and $0\le f_1\le f_2$, then $f_1$ is ignorable (assuming $f_1\in \mathcal H$). By Archimedes’ principle, it follows that the class of ignorable functions is closed under (nonnegative) scalar multiplication.

### Lemma 5.10

For every $\varepsilon > 0$, the function $f_2(x) = 1/x^{2 + \varepsilon }$ is ignorable.

### Proof

Fix $f_1\in \mathcal R\cap \mathcal H$, and let $g_1:[t_1,\infty )\rightarrow {[0,\infty )}$ be a recursive antiderivative of $f_1$. Fix $C > 1/\varepsilon$, and let

$$\begin{aligned} g(x) := g_1(x) + \frac{C}{x^{1 + \varepsilon }}\cdot \end{aligned}$$

Then

$$\begin{aligned} -g'(x) - g^2(x)&= -\left( g_1'(x) - \frac{C(1 + \varepsilon )}{x^{2 + \varepsilon }}\right) - \left( g_1^2(x) + \frac{2Cg_1(x)}{x^{1 + \varepsilon }} + \frac{C^2}{x^{2 + 2\varepsilon }}\right) \\&= f_1(x) + \frac{C}{x^{2 + \varepsilon }}\left[ 1 + \varepsilon - 2x g_1(x) - \frac{C}{x^\varepsilon }\right] . \end{aligned}$$

Since $f_1\in \mathcal H$, we have either

5.6 $$\begin{aligned} f_1(x) \le \frac{1}{4x^2} \text { for all sufficiently large} \ x, \end{aligned}$$

or

5.7 $$\begin{aligned} f_1(x) \ge \frac{1}{4x^2} \text { for all sufficiently large} \ x \end{aligned}$$

(Lemma A.2). If (5.6) holds, then Lemmas 5.4 and 5.5 automatically show that $f_1 + f_2\in \mathcal R$. So we suppose that (5.7) holds. Let $f_3,g_3$ be defined by the equations

$$\begin{aligned} f_1(x)&= \frac{1}{x^2}\left[ \frac{1}{4} + f_3(\log (x))\right] \\ g_1(x)&= \frac{1}{x}\left[ \frac{1}{2} + g_3(\log (x))\right] . \end{aligned}$$

Then $g_3:[e^{t_1},\infty ) \rightarrow [-1/2,\infty )$ is a recursive antiderivative of $f_3$. But by (5.7), we have $f_3\ge 0$. By the argument used at the end of the proof of Lemma 5.5, the limit $\lim _{x\rightarrow \infty }g_3(x)$ exists and is equal to zero. Equivalently, this means that $xg_1(x)\rightarrow 1/2$ as $x\rightarrow \infty$. Thus

$$\begin{aligned} \lim _{x\rightarrow \infty } \left[ 1 + \varepsilon - 2x g_1(x) - \frac{C}{x^\varepsilon }\right] = \varepsilon . \end{aligned}$$

Since $C > 1/\varepsilon$, this implies that

$$\begin{aligned} -g'(x) - g^2(x) \ge f_1(x) + f_2(x) \text { for all sufficiently large} x. \end{aligned}$$

By Remark 5.3, we have $f_1 + f_2\in \mathcal R$. $\square$

### Remark

Applying Lemma 5.5 repeatedly shows that for all $N\ge -1$ and $\varepsilon > 0$ the function

$$\begin{aligned} f(x)&= \left( \prod _{i = 0}^N \frac{1}{\log ^{(i)}(x)}\right) ^2\left( \frac{1}{\log ^{(N + 1)}(x)}\right) ^{2 + \varepsilon }\\&= \frac{1}{x^2\log ^2(x)\cdots \left( \log ^{(N)}(x)\right) ^2 \left( \log ^{(N + 1)}(x)\right) ^{2 + \varepsilon }} \end{aligned}$$

is ignorable.

We finish this section by providing a number of equivalent conditions to the recursive integrability of a function $f\in \mathcal H$. The following proposition should be thought of as an analogue of the Integral Test which says that a increasing function $f:{[0,\infty )}\rightarrow {[0,\infty )}$ is integrable if and only if the series $\sum _{k = 1}^\infty f(k)$ is summable. It should be noted that as with the Integral Test, the motivation here is not to determine whether a function is recursively integrable by using an equivalent condition, but rather to determine whether one of the equivalent conditions is true by determining whether the function in question is recursively integrable.

### Proposition 5.11

Suppose $f\in \mathcal H$ is nonnegative. Then for any $t\in \mathbb R$, the following are equivalent:

(A) $$f\in \mathcal R$$.
(B1) There exists a nonnegative sequence $(S_k)_{k\ge k_0}$ satisfying
  5.8 $$\begin{aligned} S_k - S_{k + 1} \ge S_{k + 1}^2 + f(k). \end{aligned}$$
(B2) There exists a nonnegative sequence $(S_k)_{k\ge k_0}$ satisfying
  5.9 $$\begin{aligned} S_k - S_{k + 1} = S_{k + 1}^2 + f(k). \end{aligned}$$
(C1) There exists a nonnegative sequence $(S_k)_{k\ge k_0}$ satisfying $S_k\rightarrow 0$ and
  5.10 $$\begin{aligned} S_k - S_{k + 1} \ge S_k^2 + t S_k^3 + f(k). \end{aligned}$$
(C2) There exists a nonnegative sequence $(S_k)_{k\ge k_0}$ satisfying $S_k\rightarrow 0$ and
  5.11 $$\begin{aligned} S_k - S_{k + 1} = S_k^2 + t S_k^3 + f(k). \end{aligned}$$

### Remark

Suppose that *f* satisfies any of the conditions (B1)–(C2). Plugging the formula $S_k\rightarrow 0$ into the appropriate Eqs. (5.8) or (5.10) shows that $\limsup _{k\rightarrow \infty } f(k) \le 0$. Since $f\in \mathcal H$ and $f\ge 0$, it follows that $f(x)\rightarrow 0$ as $f\rightarrow \infty$. Again using the facts that $f\in \mathcal H$ and $f\ge 0$, we deduce that *f* is decreasing for sufficiently large *x*. Similar reasoning applies if we assume that *f* satisfies (A).

Thus in the proof of Proposition 5.11, we may assume that *f* is decreasing on its domain of definition.

### Remark 5.12

Conditions (A), (B1), and (C1) all have the property that when $f_2$ satisfies the condition and $0\le f_1\le f_2$, then $f_1$ also satisfies the condition. Thus in proving the equivalences (A) $\Leftrightarrow \;$ (B1) $\Leftrightarrow \;$ (C1), it suffices to consider the case where

5.12 $$\begin{aligned} \frac{1}{4x^2} \le f(x) \le \frac{1}{x^2} \text { for all sufficiently large} \ x. \end{aligned}$$

Indeed, suppose that Proposition 5.11 holds whenever *f* satisfies (5.12). Then by Lemma 5.4, the function $f_-(x) = 1/(4x^2)$ satisfies (A), (B1), and (C1) while the function $f_+(x) = 1/x^2$ fails to satisfy them. Now let $f\in \mathcal H$ be arbitrary. If *f* does not satisfy (5.12), then by Lemma A.2 either $f(x) \le f_-(x)$ for all *x* sufficiently large or $f(x) \ge f_+(x)$ for all *x* sufficiently large. In the first case, (A), (B1), and (C1) hold while in the second case, (A), (B1), and (C1) fail to hold.

*Proof of (*A*)* $\Rightarrow \;$(B1). If $g:[t_1,\infty )\rightarrow {[0,\infty )}$ is a recursive antiderivative of *f*, then the sequence $S_k = g(k - 1)$ satisfies (5.8).

*Proof of (B1)* $\Rightarrow \;$(B2). Suppose that the sequence $(S_k)_{k\ge k_0}$ satisfies (5.8). For each $N\ge k_0$, let $(S_k^{(N)})_{k = k_0}^N$ be the unique sequence satisfying (5.9) for $k = k_0,\ldots ,N - 1$ and such that $S_N^{(N)} = 0$. Backwards induction shows that for each *k*, the sequence $(S_k^{(N)})_{N\ge k}$ is increasing, and $S_k^{(N)}\le S_k$ for all $N\ge k$. Let

$$\begin{aligned} \widetilde{S}_k = \lim _{N\rightarrow \infty } S_k^{(N)} \in {[0,\infty )}. \end{aligned}$$

Then the nonnegative sequence $(\widetilde{S}_k)_{k\ge k_0}$ satisfies (5.9).

*Proof of* (B2) $\Rightarrow \;$(C1). Suppose that $(S_k)_{k\ge k_0}$ satisfies (5.9), and that (5.12) holds.

### Claim 5.13

$$k S_k \rightarrow 1/2$$.

### Proof

By (5.9) and (5.12), we have

5.13 $$\begin{aligned} S_k - S_{k + 1} \ge S_{k + 1}^2 + \frac{1}{4k^2} \text { for all} \ k \ \text {sufficiently large.} \end{aligned}$$

In analogy with the proof of Lemma 5.10, for each *k* let $T_k\ge -1/2$ satisfy

$$\begin{aligned} S_k = \frac{1}{k}\left[ \frac{1}{2} + T_k\right] . \end{aligned}$$

Plugging into (5.13) gives

$$\begin{aligned} \frac{1}{k}(T_k - T_{k + 1}) + \frac{1}{k(k + 1)}\left[ \frac{1}{2} + T_{k + 1}\right] \ge \frac{1}{(k + 1)^2}\left[ T_{k + 1}^2 + T_{k + 1} + \frac{1}{4}\right] + \frac{1}{4k^2} \end{aligned}$$

and thus

$$\begin{aligned} \frac{1}{k}(T_k - T_{k + 1}) \ge \frac{T_{k + 1}^2}{(k + 1)^2}\cdot \end{aligned}$$

It follows that the sequence $(T_k)_1^\infty$ is decreasing and bounded from below. Thus the limit $\lim _{k\rightarrow \infty }T_k$ exists, and

$$\begin{aligned} \infty > \sum _k (T_k - T_{k + 1}) \ge \sum _k \frac{k}{(k + 1)^2} T_{k + 1}^2 \asymp \sum _k \frac{1}{k} T_{k + 1}^2, \end{aligned}$$

which implies that $\lim _{k\rightarrow \infty }T_k = 0$. Equivalently, $\lim _{k\rightarrow \infty } k S_k = 1/2$. $\square$

In particular, $S_k\rightarrow 0$. Fix $C > 0$, and let

$$\begin{aligned} \widetilde{S}_k = S_k + \frac{C}{k^2}\cdot \end{aligned}$$

Then $\widetilde{S}_k\rightarrow 0$ as well. So to complete the proof, we need to show that (5.10) holds for the sequence $(\widetilde{S}_k)_{k\ge k_0}$. We have

$$\begin{aligned} \widetilde{S}_k - \widetilde{S}_{k + 1}&\ge S_k - S_{k + 1} + \frac{2C}{(k + 1)^3}\\&= S_{k + 1}^2 + f(k) + \frac{2C}{(k + 1)^3}\\&= \widetilde{S}_k^2 + f(k) + \frac{2C}{(k + 1)^3} - (\widetilde{S}_k + S_{k + 1})(\widetilde{S}_k - S_{k + 1})\\&\ge \widetilde{S}_k^2 + f(k) + \frac{2C}{(k + 1)^3} - 2\left( S_k + \frac{C}{k^2}\right) \left( S_{k + 1}^2 + f(k) + \frac{C}{k^2}\right) . \end{aligned}$$

Let

$$\begin{aligned} E_k = \frac{2C}{(k + 1)^3} - 2\left( S_k + \frac{C}{k^2}\right) \left( S_{k + 1}^2 + f(k) + \frac{C}{k^2}\right) - t \widetilde{S}_k^3, \end{aligned}$$

so that

$$\begin{aligned} \widetilde{S}_k - \widetilde{S}_{k + 1} \ge \widetilde{S}_k^2 + f(k) + t \widetilde{S}_k^3 + E_k. \end{aligned}$$

So to complete the proof, it suffices to show that if *C* is large enough, then $E_k \ge 0$ for all *k* sufficiently large. And indeed,

$$\begin{aligned} \liminf _{k\rightarrow \infty } k^3 E_k&= 2C - 2\limsup _{k\rightarrow \infty }\left[ \left( k S_k + \frac{C}{k}\right) \left( k^2 S_{k + 1}^2 + k^2 f(k) + C\right) \right] \\&\quad \,- t \left( \limsup _{k\rightarrow \infty } k \widetilde{S}_k\right) ^3\\&\ge 2C - 2 (1/2)(1/4 + 1 + C) - t/8 = C - t/8 - 5/4. \end{aligned}$$

Thus by choosing $C > t/8 + 5/4$, we complete the proof.

*Proof of* (C1) $\Rightarrow \;$(C2). Suppose that the sequence $(S_k)_{k\ge k_0}$ satisfies $S_k\rightarrow 0$ and (5.10). Fix $k_1\ge k_0$ large enough so that

$$\begin{aligned} S_{k_1} \le \frac{1}{\max (5,|t| + 1)}\cdot \end{aligned}$$

Then for all $0 < x\le S_{k_1}$, we have $x^2 + tx^3 > 0$ and $(\mathrm {d}/\mathrm {d}x)[x - x^2 - tx^3] \ge 0$. Let $(\widetilde{S}_k)_{k\ge k_1}$ be the unique sequence satisfying (5.11) and $\widetilde{S}_{k_1} = S_{k_1}$. An induction argument shows that for all $k\ge k_1$, $S_k \le \widetilde{S}_k \le S_{k_1}$ and $\widetilde{S}_{k + 1} \le \widetilde{S}_k$. In particular the sequence $(\widetilde{S}_k)_{k\ge k_0}$ is nonnegative. To complete the proof we need to show that $\widetilde{S}_k\rightarrow 0$. Since $(\widetilde{S}_k)_k$ is decreasing, the limit $L = \lim _{k\rightarrow \infty }\widetilde{S}_k$ exists. Taking the limit of (5.11) we find that

$$\begin{aligned} L - L = L^2 + tL^3. \end{aligned}$$

Since $0\le L \le S_{k_1}$, this implies that $L = 0$.

*Proof of* (C1) $\Rightarrow \;$(A). First suppose $t = 0$. If $(S_k)_{k\ge k_0}$ satisfies $S_k\rightarrow 0$ and (5.10), then let *g* be the linear interpolation of $(S_k)_{k\ge k_0}$, i.e.

$$\begin{aligned} g(x) = S_k + (x - k)(S_{k + 1} - S_k) \text { for } k \le x \le k + 1. \end{aligned}$$

Then

$$\begin{aligned} -g'(x) = S_{k + 1} - S_k \ge S_k^2 + f(k) \ge g^2(x) + f(x) \;\;\forall k< x < k + 1, \end{aligned}$$

so by Remark 5.3 $f\in \mathcal R$.

Now suppose $t\ne 0$ and that $(S_k)_{k\ge k_0}$ satisfies $S_k\rightarrow 0$ and (5.10). Let $\ell$ be large enough so that

$$\begin{aligned} \frac{2}{k} - \left( \frac{2}{k}\right) ^2 - t\left( \frac{2}{k}\right) ^3 \le \frac{2}{k + 1} \;\;\forall k\ge \ell , \end{aligned}$$

and let $k_1\ge k_0$ be large enough so that $S_{k_1 + \ell } \le 2/\ell$. Then an induction argument shows that

5.14 $$\begin{aligned} S_k \le \frac{2}{k - k_1} \;\;\forall k\ge k_1 + \ell . \end{aligned}$$

In particular, there exists $C > 0$ such that $S_k \le C/k$ for all $k\ge k_0$. Then

$$\begin{aligned} S_k - S_{k + 1} \ge S_k^2 + f(k) - \frac{|t|C^3}{k^3}, \end{aligned}$$

and so by the $t = 0$ case of (C1) $\Rightarrow \;$(A), the function $x\mapsto f(x) - |t|C^3/x^3$ is recursively integrable. Since the function $x\mapsto |t| C^3/x^3$ is ignorable (Lemma 5.10), *f* is also recursively integrable.

## Proof of Theorems 1.3 and 1.4 and formula (1.2)

As in Sect. 5, $\mathcal H$ denotes a Hardy field which contains the exponential and logarithm functions and is closed under composition, for example the field of Hardy *L*-functions. As in Sect. 4, we write

$$\begin{aligned} \gamma _d&= (d - 1)^{1/d} > 1 (\text {if }d\ge 3)\\ \alpha _d&= \gamma _d + \gamma _d^{-(d - 1)} = d(d - 1)^{-(d - 1)/d}. \end{aligned}$$

Theorems 1.3 and 1.4 and formula (1.2) will all follow from the following result:

### Proposition 6.1

Suppose that $d\ge 3$, and fix $\psi \in \mathcal H$. Then the following are equivalent:

(A) $\psi$ is Dirichlet on $\mathbb R^d$ with respect to $H_{\mathtt{max}}$.
(B) $\psi$ is uniformly Dirichlet on $\mathbb R^d$ with respect to $H_{\mathtt{max}}$.
(C) $C_{H_{\mathtt{max}},\psi }(\mathbf x) = 0$ for all $\mathbf x\in \mathbb R^d$, i.e. $\psi$ is non-optimally Dirichlet on $\mathbb R^d$ with respect to $H_{\mathtt{max}}$.
(D) The function
  $$\begin{aligned} f_\psi (x) = \frac{2}{d\gamma _d}\left[ \alpha _d + \frac{\log \psi (e^{\gamma _d^x})}{\gamma _d^x}\right] \end{aligned}$$
  is nonnegative for large values of *x* and satisfies $f_\psi \notin \mathcal R$.

In particular, no function $\psi \in \mathcal H$ is optimally Dirichlet on $\mathbb R^d$ with respect to the height function $H_{\mathtt{max}}$.

### Proof of Theorems 1.3 and 1.4 and formula 1.2 assuming Proposition 6.1

Suppose that Proposition 6.1 is true. Then for all $\alpha \ge 0$, $\psi _\alpha$ is Dirichlet on $\mathbb R^d$ with respect to $H_{\mathtt{max}}$ if and only if $\alpha < \alpha _d$. It follows that $\omega _d(H_{\mathtt{max}}) = \alpha _d$, demonstrating the formula (1.2).

Since Theorem 1.3 is a restatement of the equivalence of (A) and (C) of Proposition 6.1, to complete the proof it suffices to prove Theorem 1.4. Specifically, given $N\ge 1$ and $C\ge 0$, we must show that the function $\psi _{N,C}$ of Theorem 1.4 satisfies the equivalent conditions (A)-(D) of Proposition 6.1 if and only if $C > 1$. Considering condition (D), we must show that $f_{\psi _{N,C}}\in \mathcal R$ if and only if $C\le 1$. But

$$\begin{aligned} f_{\psi _{N,C}}(x) = \log ^2(\gamma _d) f_{N - 2,C/4}(x\log (\gamma _d)), \end{aligned}$$

so this follows from Corollary 5.6 and Lemma 5.8. $\square$

The proof of Proposition 6.1 will be divided into three parts: the proof of (D) $\Rightarrow \;$(B), which constitutes the hardest part of the argument; the proof of (C) $\Rightarrow \;$(D), which is essentially the proof of (D) $\Rightarrow \;$(B) in reverse, but made easier due to the explicitness of the data structure in question; and finally, the reduction of the theorem to those two implications, which is essentially a corollary of Lemma 5.10.

### Remark

Throughout the proof we will assume that

6.1 $$\begin{aligned} \frac{1}{4x^2} \le f_\psi (x) \le \frac{1}{x^2} \text { for all} \ x \ \text {sufficiently large.} \end{aligned}$$

The justification of this assumption follows along the same lines as Remark 5.12. Specifically, suppose that Proposition 6.1 holds whenever $\psi$ satisfies (6.1). Let $\psi _-$ and $\psi _+$ denote the functions for which equality holds in the left and right hand inequalities of (6.1), respectively. Then by Lemma 5.4, $\psi _+$ satisfies (A)-(D) of Proposition 6.1 while $\psi _-$ fails to satisfy them. Now let $\psi \in \mathcal H$ be arbitrary. If $\psi$ does not satisfy (6.1), then by Lemma A.2 either $\psi (q) \ge \psi _+(q)$ for all *q* sufficiently large or $\psi (q) \le \psi _-(q)$ for all *q* sufficiently large. In the first case, we have $C_{H_{\mathtt{max}},\psi }\le C_{H_{\mathtt{max}},\psi _+}$ and so (A)-(D) of Proposition 6.1 hold. In the second case, we have $C_{H_{\mathtt{max}},\psi } \ge C_{H_{\mathtt{max}},\psi _-}$ and so (A)-(D) of Proposition 6.1 fail to hold.

### Remark 6.2

When reading the proof of (D) $\Rightarrow \;$(B), one should check that the implications (6.3) $\Rightarrow \;$(6.4) $\Rightarrow \;$(6.5) $\Rightarrow \;$(6.10) are all invertible if one assumes the following facts about $\Delta$: $\max (\Delta _k) = A_k$ for all $k\in \mathbb N$, and $\Delta$ is eventually periodic in the sense of Claim 6.6. The converse directions will be used in the proof of (C) $\Rightarrow \;$(D).

**Notation** The following notations will be used in the course of the proof:

$$\begin{aligned} \Psi (b)&= -\log \psi (e^b)\\ \Phi (b)&= \alpha _d - \frac{\Psi (b)}{b}\cdot \end{aligned}$$

Note that according to these notations,

6.2 $$\begin{aligned} f_\psi (x) = \frac{2}{d\gamma _d} \Phi (\gamma _d^x). \end{aligned}$$

### Proof of (D) $\Rightarrow \;$(B)

We prove the contrapositive. Suppose that $\sup _{\mathbb R^d{\setminus }\mathbb Q^d} C_{H_{\mathtt{max}},\psi } = \infty$, and we will show that $f_\psi \in \mathcal R$. By Lemma 2.4, we have $\sup _\Delta C_{\mathtt{max},\Psi }(\Delta ) = \infty$, where the supremum is taken over *d*-dimensional data progressions $\Delta$. In particular, there exists a *d*-dimensional data progression $\Delta = (A_k,i_k)_{k = 1}^\infty$ such that $C_{\mathtt{max},\Psi }(\Delta ) > 0$. It follows that

6.3 $$\begin{aligned} b_k^{(i_k)} + b_{k + 1}^{(i_k)} \le \Psi (\max (\Delta _k)) \end{aligned}$$

for all *k* sufficiently large.

#### Claim 6.3

We may suppose without loss of generality that $\max (\Delta _k) = A_k$ for all $k\in \mathbb N$.

#### Proof

Consider the set $K = \{k\in \mathbb N: \max (\Delta _{k + 1}) > \max (\Delta _k)\}$. The set *K* is infinite by part (II) of the definition of a data progression. Let $(k_\ell )_1^\infty$ be the unique increasing indexing of *K*, and consider the data progression $\widetilde{\Delta }= (\max (\Delta _{k_\ell }),i_{k_\ell })_{\ell = 1}^\infty$. Note that for all $\ell \in \mathbb N$ and $i = 1,\ldots ,d$,

$$\begin{aligned} \widetilde{b}_\ell ^{(i)}&\le b_{k_\ell }^{(i)}\\ \max (\widetilde{\Delta }_\ell )&= \widetilde{A}_\ell = \max (\Delta _{k_\ell }). \end{aligned}$$

Moreover, if $k = k_\ell$, then

$$\begin{aligned} b_{k + 1}^{(i_k)} = \max (\Delta _{k + 1}) = \max (\Delta _{k_{\ell + 1}}) = A_{\ell + 1} = \widetilde{b}_{\ell + 1}^{(\widetilde{i}_\ell )}. \end{aligned}$$

Plugging all these into (6.3) gives

$$\begin{aligned} \widetilde{b}_\ell ^{(\widetilde{i}_\ell )} + \widetilde{b}_{\ell + 1}^{(\widetilde{i}_\ell )} \le \Psi (\max (\widetilde{\Delta }_\ell )), \end{aligned}$$

i.e. (6.3) holds for the data progression $\widetilde{\Delta }$. $\square$

So in what follows, we assume that $\max (\Delta _k) = A_k$ for all $k\in \mathbb N$. Using this fact together with (2.6), (6.3) becomes

$$\begin{aligned} b_k^{(i_k)} \le \Psi (A_k) - A_{k + 1}. \end{aligned}$$

Letting $t_k = A_{k + 1}/A_k$, we may rewrite the above equation as

6.4 $$\begin{aligned} b_k^{(i_k)} \le A_k(\alpha _d - \Phi (A_k) - t_k). \end{aligned}$$

For each $k\in \mathbb N$ let

$$\begin{aligned} f_k = \frac{\prod _{i = 1}^d b_k^{(i)}}{(A_k)^d}; \end{aligned}$$

using (2.6), (6.4) then becomes

6.5 $$\begin{aligned} \frac{f_k}{f_{k + 1}} \le (\alpha _d - \Phi (A_k) - t_k) t_k^{d - 1}. \end{aligned}$$

#### Claim 6.4

For some $k_1\in \mathbb N$, the sequence $(f_k)_{k_1}^\infty$ is increasing.

#### Proof

By (6.1), we have $\Phi (b) \ge 0$ for all *b* sufficiently large. Thus by Corollary 4.4,

6.6 $$\begin{aligned} \frac{f_k}{f_{k + 1}} \le (\alpha _d - t_k) t_k^{d - 1} \le 1 \end{aligned}$$

for all *k* sufficiently large. $\square$

#### Claim 6.5

$$t_k \rightarrow \gamma _d$$ as $$k\rightarrow \infty $$.

#### Proof

We clearly have $f_k\le 1$ for all *k*, so by Claim 6.4, the sequence $(f_k)_1^\infty$ converges to a positive number. Thus $\frac{f_k}{f_{k + 1}} \rightarrow 1$. Combining with (6.6), we see that $(\alpha _d - t_k) t_k^{d - 1} \rightarrow 1$. Applying Corollary 4.4 again, we get $t_k \rightarrow \gamma _d$. $\square$

#### Claim 6.6

$\Delta$ is eventually periodic in the following sense: there exists a permutation $\sigma :\{1,\ldots ,d\}\rightarrow \{1,\ldots ,d\}$ such that for all *k* sufficiently large,

6.7 $$\begin{aligned} i_k = \sigma (j_k) \text { where }j_k = k \text { (mod { d})}. \end{aligned}$$

#### Proof

Combining (6.4) and Claim 6.5, we see that

$$\begin{aligned} \limsup _{k\rightarrow \infty } \frac{b_k^{(i_k)}}{A_k} \le \alpha _d - \gamma _d = \gamma _d^{-(d - 1)}. \end{aligned}$$

On the other hand, for each $j = 0,\ldots ,d - 2$, by Claim 6.5 we have

$$\begin{aligned} \lim _{k\rightarrow \infty } \frac{A_{k - j}}{A_k} = \gamma _d^{-j} > \gamma _d^{-(d - 1)}. \end{aligned}$$

It follows that $b_k^{(i_k)} = A_{\ell (i_k,k) + 1} \ne A_{k - j}$ for all *k* sufficiently large. In particular $\ell (i_k,k) \ne k - j - 1$. Now fix $k_2\in \mathbb N$ such that for all $k\ge k_2$ and $j = 0,\ldots ,d - 2$, we have $\ell (i_k,k) \ne k - j - 1$. Then $\ell (i_k,k) \le k - d$, so $i_{k - j} \ne i_k$ for all $j = 1,\ldots ,d - 1$. In particular, the sets

$$\begin{aligned} \{i_k,\ldots ,i_{k + d - 1}\} \text { and } \{i_{k + 1},\ldots ,i_{k + d}\} \end{aligned}$$

both contain *d* distinct elements. It follows that $i_k = i_{k + d}$, so the sequence $(i_k)_{k\ge k_2}$ is periodic of period *d*. At this point, it is clear that (6.7) holds for some permutation $\sigma$. $\square$

#### Corollary 6.7

For all sufficiently large *k*,

6.8 $$\begin{aligned} f_k = \prod _{j = 1}^{d - 1} \frac{A_{k - j}}{A_k} = \prod _{j = 1}^{d - 1}\frac{1}{t_{k - j}^{d - j}}\cdot \end{aligned}$$

#### Proof

Fix *k* large enough such that the set $\{i_{k - d},\ldots ,i_{k - 1}\}$ contains *d* distinct elements; this is possible by Claim 6.6. It follows that

$$\begin{aligned} \{\ell (i,k) : i = 1,\ldots ,d\} = \{k - 1,\ldots ,k - d\} \end{aligned}$$

and thus

$$\begin{aligned} \prod _{i = 1}^d b_k^{(i)} = \prod _{i = 1}^d A_{\ell (i,k) + 1} = \prod _{j = 1}^d A_{k - j + 1}. \end{aligned}$$

Dividing both sides by $(A_k)^d$ finishes the proof.

#### Corollary 6.8

For all *k*,

6.9 $$\begin{aligned} A_k \gtrsim \gamma _d^k. \end{aligned}$$

#### Proof

By Claim 6.5,

$$\begin{aligned} f_k \xrightarrow [k]{} \prod _{j = 1}^{d - 1} \frac{1}{\gamma _d^{d - j}} = \gamma _d^{-\left( {\begin{array}{c}d\\ 2\end{array}}\right) }. \end{aligned}$$

By Claim 6.4, it follows that $$f_k \le \gamma _d^{-\left( {\begin{array}{c}d\\ 2\end{array}}\right) }$$ for all *k* sufficiently large. Let $k_3$ be large enough so that (6.8) holds for all $k\ge k_3$; then

$$\begin{aligned} \gamma _d^{-k\left( {\begin{array}{c}d\\ 2\end{array}}\right) } \gtrsim \prod _{\ell = k_3}^{k - 1} f_\ell = \prod _{j = 1}^{d - 1}\prod _{\ell = k_3}^{k - 1}\frac{1}{t_{\ell - j}^{d - j}} = \prod _{j = 1}^{d - 1}\left( \frac{A_{k_3 - j}}{A_{k - j}}\right) ^{d - j}, \end{aligned}$$

and thus

$$\begin{aligned} A_k^{\left( {\begin{array}{c}d\\ 2\end{array}}\right) } \ge \prod _{j = 1}^{d - 1} (A_{k - j})^{d - j} \gtrsim \gamma _d^{k\left( {\begin{array}{c}d\\ 2\end{array}}\right) }. \end{aligned}$$

Taking $$\left( {\begin{array}{c}d\\ 2\end{array}}\right) $$th roots completes the proof. $\square$

Using Corollary (6.7), (6.5) becomes

$$\begin{aligned} \prod _{j = 1}^{d - 1}\frac{t_k}{t_{k - j}} \le (\alpha _d - \Phi (A_k) - t_k)t_k^{d - 1}, \end{aligned}$$

or equivalently

$$\begin{aligned} t_k \le \alpha _d - \Phi (A_k) - \prod _{j = 1}^{d - 1}\frac{1}{t_{k - j}}\cdot \end{aligned}$$

Writing $s_k = t_k/\gamma _d - 1$, a few arithmetic calculations show that the above inequality is equivalent to

6.10 $$\begin{aligned} s_k \le \frac{1}{d - 1}\left[ 1 - \prod _{j = 1}^{d - 1}\frac{1}{1 + s_{k - j}}\right] - \frac{\Phi (A_k)}{\gamma _d}\cdot \end{aligned}$$

Consequently, it becomes important to study behavior the function

$$\begin{aligned} f(x_1,\ldots ,x_{d - 1}) = 1 - \prod _{j = 1}^{d - 1}\frac{1}{1 + x_j} \end{aligned}$$

near the origin. We calculate the gradient and Hessian of *f* at $\mathbf 0$:

$$\begin{aligned} f'(\mathbf 0)&= \sum _{j = 1}^{d - 1} \mathbf e_j\\ f''(\mathbf 0)&= -\left[ \sum _{j = 1}^{d - 1} \mathbf e_j^2 + \left( \sum _{j = 1}^{d - 1}\mathbf e_j\right) ^2\right] \end{aligned}$$

Since $f(\mathbf 0) = 0$, this means that *f* can be estimated in a neighborhood of the origin by the formula

6.11 $$\begin{aligned} f(\mathbf x) = \sum _{j = 1}^{d - 1} x_j - \frac{1}{2}\left[ \sum _{j = 1}^{d - 1} x_j^2 + \left( \sum _{j = 1}^{d - 1}x_j\right) ^2\right] + O(\Vert \mathbf x\Vert ^3). \end{aligned}$$

In fact, we can be explicit: (6.11) holds whenever $\Vert \mathbf x\Vert \le 1/2$.

Continuing with the proof, for $k\in \mathbb N$ let

$$\begin{aligned} \phi _k = \frac{2}{d\gamma _d}\Phi (A_k) \end{aligned}$$

(cf. (6.2)).

#### Claim 6.9

For all *k* sufficiently large,

$$\begin{aligned} |\phi _{k + 1} - \phi _k| \lesssim \frac{1}{k^3}\cdot \end{aligned}$$

#### Proof

Since $f_\psi \in \mathcal H$, we may differentiate the inequalities (6.1) (cf. Lemma A.3) to get

6.12 $$\begin{aligned} |f_\psi '(x)| \le \left| \frac{\mathrm {d}}{\mathrm {d}x}\left[ \frac{1}{x^2}\right] \right| = \frac{2}{x^3} \text { for all}\ x \ \text {sufficiently large.} \end{aligned}$$

Using (6.2) and applying the fundamental theorem of calculus, we have

$$\begin{aligned} |\phi _{k + 1} - \phi _k|&= |f_\psi (\log _{\gamma _d}(A_{k + 1})) - f_\psi (\log _{\gamma _d}(A_k))| \\&\le \frac{2}{\log _{\gamma _d}^3(A_k)} \log _{\gamma _d}(t_k) \\&\lesssim \frac{1}{k^3}\cdot ({ by \ Claim} \ 6.5 \ { and}\ { Corollary} \ 6.8)&\end{aligned}$$

$$\square $$

Now fix $C_1 > 0$ large to be determined, then fix $\delta > 0$ small to be determined (possibly depending on $C_1$), and finally fix $k_0\in \mathbb N$ large to be determined (possibly depending on both $\delta$ and $C_1$). Let $(S_k)_{k = k_0}^\infty$ be the unique sequence defined by the equations

6.13 $$\begin{aligned} S_{k + 1} = S_k - S_k^2 - \phi _k + \frac{C_1}{k^3} + C_1 |S_k|^3, \;\;\; S_{k_0} = \delta . \end{aligned}$$

The following claim is the heart of the proof:

#### Claim 6.10

If $k_0$ and $C_1$ are sufficiently large and $\delta$ is sufficiently small (with $k_0$ allowed to depend on $\delta$, which is in turn allowed to depend on $C_1$), then

6.14 $$\begin{aligned} -\frac{1}{\max (2,C_1)} \le s_k \le S_k \le \delta \le \frac{1}{\max (2,C_1)} \end{aligned}$$

for all $$k\ge k_0$$.

#### Proof

Throughout the proof, we will assume that $\delta < 1/\max (2,C_1)$ and that $k_0\ge 4C_1$. Since $\delta$ and $k_0$ are both allowed to depend on $C_1$, these assumptions are justified. In particular, the rightmost inequality of (6.14) requires no proof.

By Claim 6.5, we have $s_k\rightarrow 0$. Thus, the leftmost inequality of (6.14) can be achieved simply by an appropriate choice of $k_0$.

The proof of the two middle inequalities of (6.14) is by strong induction on *k*.

**Base Case:** $k = k_0,\ldots ,k_0 + d - 2$. For this part of the proof, we’ll think of $C_1,\delta > 0$ as being fixed. Define the sequence $(T_j)_{j = 0}^{d - 2}$ via the formula

$$\begin{aligned} T_{j + 1} = T_j - T_j^2 + C_1 |T_j|^3, \;\; T_0 = \delta . \end{aligned}$$

Since $\delta < 1/\max (2,C_1)$, the sequence $(T_j)_{j = 0}^{d - 2}$ is strictly decreasing and strictly positive. Note that for each $j = 0,\ldots ,d - 2$,

$$\begin{aligned} S_{k_0 + j}^{(k_0)} \xrightarrow [k_0]{} T_j, \end{aligned}$$

where the superscript of $k_0$ is merely making explicit the fact that the sequence $(S_k)_{k\ge k_0}$ depends on $k_0$. On the other hand,

$$\begin{aligned} s_{k_0 + j} \xrightarrow [k_0]{} 0 < T_j. \end{aligned}$$

So if $k_0$ is sufficiently large, then (6.14) holds for $k = k_0 + j$.

**Inductive Step:** Fix $\ell \ge k_0 + d - 1$, and suppose that (6.14) holds for $k = \ell - d + 1,\ldots ,\ell - 1$. We claim that (6.14) holds for $k = \ell$. $\square$

#### Subclaim 6.11

For $$j = 1,\ldots ,d - 1$$,

$$\begin{aligned} S_{\ell - j + 1} \le S_{\ell - j}. \end{aligned}$$

#### Proof

By (6.1), we have

$$\begin{aligned} \phi _k \ge \frac{1}{4k^2}\cdot \end{aligned}$$

Since $k_0\ge 4C_1$, combining with (6.13) gives

6.15 $$\begin{aligned} S_{k + 1} \le S_k - S_k^2 + C_1 |S_k|^3 \;\;\forall k\ge k_0. \end{aligned}$$

Plugging in $k = \ell - j$, we have $|S_k| \le 1/C_1$ by the induction hypothesis. Thus $S_{k + 1} \le S_k$.

In particular, plugging in $j = 1$ and using the induction hypothesis, we see that the third inequality of (6.14) holds for $k = \ell$. So to complete the proof, we need only to demonstrate that the second inequality of (6.14) holds for $k = \ell$.

#### Subclaim 6.12

For $$j = 1,\ldots ,d - 1$$,

$$\begin{aligned} |S_{\ell - j}|&\lesssim \max (1/\ell ^2,|S_{\ell - j + 1}|)\\ |S_{\ell - j + 1}|&\lesssim \max (1/\ell ^2,|S_{\ell - j}|)\\ \end{aligned}$$

#### Remark

We emphasize that here and below, the implied constants of asymptotics may not depend on $C_1$, $\delta$, or $k_0$.

#### Proof

By (6.1), we have

$$\begin{aligned} \phi _k \le \frac{1}{k^2}\cdot \end{aligned}$$

On the other hand, since $k_0\ge C_1$ we have $C_1/k^3 \le 1/k^2$ for all $k\ge k_0$. Letting $k = \ell - j$, combining with (6.13), and writing $x = S_{\ell - j}$, $y = S_{\ell - j + 1}$, we have

$$\begin{aligned} \left| x - x^2 + C_1|x|^3 - y\right| \lesssim \frac{1}{(\ell - j)^2} \asymp \frac{1}{\ell ^2}\cdot \end{aligned}$$

By the induction hypothesis, we have

6.16 $$\begin{aligned} |x|\le 1/\max (2,C_1). \end{aligned}$$

It follows that

$$\begin{aligned} |y| \lesssim \max (1/\ell ^2, |x - x^2 + C_1|x|^3|) \lesssim \max (1/\ell ^2,|x|). \end{aligned}$$

On the other hand, (6.16) also implies that $x - x^2 + C_1|x|^3 \le x$. In particular, if *x* is negative then

$$\begin{aligned} |x| \le \left| x - x^2 + C_1|x|^3\right| \lesssim \max (1/\ell ^2,|y|). \end{aligned}$$

Finally, if *x* is positive, then we have

$$\begin{aligned} |x| = x \asymp x - x^2 \le x - x^2 + C_1|x|^3 \lesssim \max (1/\ell ^2,|y|). \end{aligned}$$

$$\square $$

#### Subclaim 6.13

Let

$$\begin{aligned} a_\ell = \max \left( \frac{1}{\ell },|S_\ell |\right) . \end{aligned}$$

Then $$a_\ell \lesssim 1/C_1$$.

#### Proof

Since $\ell \ge k_0 \ge C_1$, we have $1/\ell \le 1/C_1$. On the other hand, by Subclaim 6.12 and the induction hypothesis we have

$$\begin{aligned} |S_\ell | \lesssim \max \left( \frac{1}{\ell ^2},|S_{\ell - 1}|\right) \le \frac{1}{C_1}\cdot \end{aligned}$$

$$\square $$

#### Definition 6.14

For the purposes of this proof, an expression will be called *negligible* if its absolute value is less than a constant times $a_\ell ^3$. (The constant must be independent of $C_1$, $\delta$, and $k_0$.) We’ll write $A\sim B$ if the difference between two expressions *A* and *B* is negligible.

Note that by Subclaim 6.12, we have $|S_{\ell - j}| \lesssim a_\ell$ for all $j = 0,\ldots ,d - 1$. It follows from this and (6.13) (keeping in mind Subclaim 6.13 and Claim 6.9) that $|S_{\ell - j + 1} - S_{\ell - j}| \lesssim a_\ell ^2$, and thus that

$$\begin{aligned} S_{\ell - j_1} (S_{\ell - j_2} - S_{\ell - j_2 + 1}) \sim 0 \end{aligned}$$

for all $j_1 = 0,\ldots ,d - 1$ and $j_2 = 1,\ldots ,d - 1$. It follows that

$$\begin{aligned} S_{\ell - j_1} S_{\ell - j_2} \sim S_\ell ^2 \end{aligned}$$

for all $$j_1,j_2 = 0,\ldots ,d - 1$$.

We are now ready to continue our calculation:

$$\begin{aligned} s_\ell&\le \frac{1}{d - 1}f(S_{\ell - d + 1},\ldots ,S_{\ell - 1}) - \frac{d}{2} \phi _\ell \ (\mathrm{by}\ (6.10))\\&\sim \frac{1}{d - 1}\left[ \sum _{j = 1}^{d - 1} S_{\ell - j} - \frac{1}{2} \left[ \sum _{j = 1}^{d - 1} S_\ell ^2 + \left( \sum _{j = 1}^{d - 1} S_\ell \right) ^2\right] \right] - \frac{d}{2} \phi _\ell \ (\mathrm{by}\ (6.11))\\&= \frac{1}{d - 1}\left[ \sum _{j = 1}^{d - 1} S_{\ell - j} - \left( {\begin{array}{c}d\\ 2\end{array}}\right) S_\ell ^2\right] - \frac{d}{2} \phi _\ell \\ \end{aligned}$$

$$\begin{aligned} \sum _{j = 1}^{d - 1} [S_{\ell - j} - S_\ell ]&= \sum _{j = 1}^{d - 1}\sum _{i = 1}^j \left[ S_{\ell - i}^2 + \phi _{\ell - i} - C_1 \left[ \frac{1}{(\ell - i)^3} + S_{\ell - i}^3\right] \right] (\mathrm{by} \ (6.13))\\&\sim \sum _{j = 1}^{d - 1}\sum _{i = 1}^j \left[ S_\ell ^2 + \phi _\ell - C_1 \left[ \frac{1}{\ell ^3} + |S_\ell |^3\right] \right] \\&= \left( {\begin{array}{c}d\\ 2\end{array}}\right) \left[ S_\ell ^2 + \phi _\ell - C_1 \left[ \frac{1}{\ell ^3} + |S_\ell |^3\right] \right] \\ s_\ell - S_\ell&\le \frac{1}{d - 1}f(S_{\ell - d + 1},\ldots ,S_{\ell - 1}) - \frac{d}{2} \phi _\ell - S_\ell \\&\sim \frac{d}{2}\left[ \phi _\ell - C_1 \left[ \frac{1}{\ell ^3} + |S_\ell |^3\right] \right] - \frac{d}{2} \phi _\ell \\&= -\frac{d}{2} C_1 \left[ \frac{1}{\ell ^3} + |S_\ell |^3\right] \le - \frac{d}{2} C_1 a_\ell ^3 \end{aligned}$$

By the definition of negligibility, we have

$$\begin{aligned} s_\ell - S_\ell \le C_2 a_\ell ^3 - \frac{d}{2} C_1 a_\ell ^3 \end{aligned}$$

for some constant $C_2$ independent of $C_1$, $\delta$, and $k_0$. By letting $C_1 = (2/d)C_2$, we have $s_\ell \le S_\ell$, completing the proof.

Having finished the proof of Claim 6.10, we continue with the proof of Proposition 6.1 (D) $\Rightarrow \;$(B). Since $S_k\ge s_k\rightarrow 0$ and since the sequence $(S_k)_{k\ge k_0}$ is decreasing by Subclaim 6.11, we have $S_k \ge 0$ for all $k\ge k_0$. The proof of Proposition 5.11 (C1) $\Rightarrow \;$(A) now shows that there exists $C_3 > 0$ such that $S_k \le C_3/k$ for all $k\ge k_0$ (cf. (5.14)). Combining with (6.14), we see that

$$\begin{aligned} A_k&= A_{k_0}\prod _{\ell = k_0}^{k - 1} \gamma _d(1 + s_\ell ) \le A_{k_0}\gamma _d^{k - k_0} \prod _{\ell = k_0}^{k - 1} (1 + C_3/\ell )= A_{k_0} \gamma _d^{k - k_0}\prod _{\ell = k_0}^{k - 1} \frac{\ell + C_3}{\ell } \\&\le C_4 \gamma _d^k k^n, \end{aligned}$$

where $n = \lceil C_3\rceil$ and $C_4 > 0$. So for all sufficiently large *k*,

$$\begin{aligned} \phi _k \ge \frac{2}{d\gamma _d} \Phi (C_4 \gamma _d^k k^n). \end{aligned}$$

Applying the fundamental theorem of calculus to (6.12) gives

$$\begin{aligned} f_\psi (k) - \phi _k&\le f_\psi (k) - \frac{2}{d\gamma _d}\Phi (C_4 \gamma _d^k k^n)\\&= f_\psi (k) - f_\psi \left( k + \log _{\gamma _d}(C_4k^n)\right) \\&\le \frac{2}{k^3} \log _{\gamma _d}(C_4k^n) \asymp \frac{\log (k)}{k^3}\cdot \end{aligned}$$

Let $C_5 > 0$ be the implied constant. Combining with (6.13) shows that

$$\begin{aligned} S_k - S_{k + 1} \ge S_k^2 - C_1 S_k^3 + f_\psi (k) - \frac{C_1}{k^3} - \frac{C_5 \log (k)}{k^3} \end{aligned}$$

for all sufficiently large *k*. By Proposition 5.11, the function

$$\begin{aligned} x\mapsto f_\psi (x) - \frac{C_1}{x^3} - \frac{C_5 \log (x)}{x^3} \end{aligned}$$

is recursively integrable. By Lemma 5.10, it follows that $f_\psi \in \mathcal R$.

### Proof of (C) $\Rightarrow \;$(D)

As before, we will prove the contrapositive. Suppose that $f_\psi \in \mathcal R$, and we will show that $\sup _{\mathbb R^d{\setminus }\mathbb Q^d} C_{H_{\mathtt{max}},\psi } > 0$. Fix $C_1 > 0$ large to be determined. By Lemma 5.10, the function $x\mapsto f_\psi (x) + C_1/x^3$ is recursively integrable. Thus by Proposition 5.11, there exists a nonnegative sequence $(S_k)_{k\ge k_0}$ satisfying

6.17 $$\begin{aligned} S_{k + 1} = S_k - S_k^2 - C_1S_k^3 - f_\psi (k) - \frac{C_1}{k^3} \;\;\forall k\ge k_0. \end{aligned}$$

For $k\ge k_0$, let $s_k = S_k$, $t_k = \gamma _d(1 + s_k)$, and

$$\begin{aligned} A_k = \gamma _d^{k_0} \prod _{j = k_0}^{k - 1} t_j = \gamma _d^k \prod _{j = k_0}^{k - 1}(1 + s_j). \end{aligned}$$

Let $i_k = k$ (mod *d*), and consider the *d*-dimensional data progression $\Delta = (A_k,i_k)_{k = k_0}^\infty$. Since the sequence $(A_k)_{k_0}^\infty$ is increasing, Remark 6.2 applies and we have the implication (6.10) $\Rightarrow \;$(6.3). Note that if (6.3) holds for all *k* sufficiently large, then we are done, as $C_{\mathtt{max},\Psi }(\Delta ) \ge 0$ and then Lemma 2.4 completes the proof.

Let us proceed to demonstrate (6.10). We begin by reproving Subclaims 6.11, 6.12, and 6.13 in our new context. Fix $k\in \mathbb N$. The inequality $S_{k + 1} \le S_k$ is immediate from (6.17). If *k* is sufficiently large, then $f_\psi (k)\le 1/k^2$, $k\ge C_1$, and $S_k\le 1/C_1$, so

$$\begin{aligned} S_k - 2S_k^2 \le S_{k + 1} + \frac{2}{k^2}\cdot \end{aligned}$$

This implies that $S_k \lesssim \max (1/k^2,S_{k + 1})$, completing the proof of the analogue of Subclaim 6.12. Finally, let $a_k = \max (1/k,S_k)$; it is immediate that $a_k \le 1/C_1$ if *k* is sufficiently large.

As in the proof of Claim 6.10 we call an expression *A* *negligible* if $|A| \lesssim a_k^3$, and write $A\sim B$ if $A - B$ is negligible. The argument following Definition 6.14 shows that $S_{k - j_1} S_{k - j_2} \sim S_k^2$ for all $j_1,j_2 = 0,\ldots ,d - 1$. Finally, the calculations on pages 25-26 can be modified to show that

$$\begin{aligned} \frac{1}{d - 1}f(s_{k - d + 1},\ldots ,s_{k - 1}) - \frac{d}{2}f_\psi (k) - s_k \sim \frac{d}{2} C_1 \left[ \frac{1}{k^3} + S_k^3\right] \ge \frac{d}{2} C_1 a_k^3. \end{aligned}$$

(Just multiply $C_1$ by $-1$ in each corresponding expression, and use $f_\psi (k)$ in place of $\phi _k$.) By the definition of negligibility, we have

$$\begin{aligned} \frac{1}{d - 1}f(s_{k - d + 1},\ldots ,s_{k - 1}) - \frac{d}{2}f_\psi (k) - s_k \ge \frac{d}{2}C_1 a_k^3 - C_2 a_k^3 \end{aligned}$$

for some constant $C_2 > 0$ independent of $C_1$. Letting $C_1 = (2/d)C_2$, we have

$$\begin{aligned} s_k \le \frac{1}{d - 1}f(s_{k - d + 1},\ldots ,s_{k - 1}) - \frac{d}{2}f_\psi (k). \end{aligned}$$

But since $A_k \ge \gamma _d^k$, we have $f_\psi (k) \ge \frac{2}{d\gamma _d}\Phi (A_k)$ for all sufficiently large *k*. Combining this inequality with the equation on the previous line gives (6.10), completing the proof.

### Completion of the proof of Proposition 6.1

Using the implications (C) $\Rightarrow \;$(D) $\Rightarrow \;$(B), we now complete the proof of Proposition 6.1. As the implications (C) $\Rightarrow \;$(B) $\Rightarrow \;$(A) are obvious, it suffices to prove that (A) $\Rightarrow \;$(D) $\Rightarrow \;$(C). Let

$$\begin{aligned} \phi (q)&= q^{1/\log ^3\log (q)}\\ g_\phi (x)&= \frac{2}{d\gamma _d} \frac{\log \phi (e^{\gamma _d^x})}{\gamma _d^x} = \frac{2}{d\gamma _d\log ^3(\gamma _d)}\frac{1}{x^3} , \end{aligned}$$

so that

$$\begin{aligned} f_{\phi \psi }&= f_\psi + g_\phi \\ f_{\psi /\phi }&= f_\psi - g_\phi . \end{aligned}$$

Since the function $g_\phi$ is ignorable, we have $f_{\phi \psi }\in \mathcal R\Leftrightarrow f_\psi \in \mathcal R\Leftrightarrow f_{\psi /\phi }\in \mathcal R$. On the other hand, $\phi (q) \rightarrow \infty$ as $q\rightarrow \infty$. Thus

$$\begin{aligned} \text {(A)} \Rightarrow \text {(C)}_{\psi = \phi \psi } \Rightarrow \text {(D)}_{\psi = \phi \psi } \Leftrightarrow \text {(D)} \Leftrightarrow \text {(D)}_{\psi = \psi /\phi } \Rightarrow \text {(B)}_{\psi = \psi /\phi } \Rightarrow \text {(C)}. \end{aligned}$$

## Open questions

In this paper, we consider only “everywhere” questions—that is, we are interested in functions $\psi$ for which $C_{H,\psi }(\mathbf x) < \infty$ for every point $\mathbf x\in \mathbb R^d{\setminus }\mathbb Q^d$. The same questions can be asked if “every” is replaced by “almost every”—with respect to Lebesgue measure or even with respect to some fractal measure. Once we know what “almost every” point does, it can be asked what is the Hausdorff dimension of the set of exceptions, i.e. the set of $\mathbf x$ which behave differently from almost every point. In the case of the height function $H_{\mathtt{lcm}}$, such questions have been extensively studied. Thus, the next step in producing a Diophantine theory of the height functions $H_{\mathtt{max}}$, $H_{\mathtt{min}}$, and $H_{\mathtt{prod}}$ similar to that for $H_{\mathtt{lcm}}$ would be to answer the following questions:

### Question 7.1

(Analogue of Khinchin’s theorem) Fix $\Theta \in \{\mathtt{max},\mathtt{min},\mathtt{prod}\}$, and let $\psi$ be a Hardy *L*-function. Must the sets $\{\mathbf x\in \mathbb R^d : C_{H_\Theta ,\psi }(\mathbf x) = 0\}$ and $\{\mathbf x\in \mathbb R^d : C_{H_\Theta ,\psi }(\mathbf x) < \infty \}$ be either null sets or full measure sets? If so, which one? Can the same theorem be proven with a weaker assumption than $\psi$ being a Hardy *L*-function (for example, assuming only that $\psi$ is decreasing)?

### Question 7.2

(Analogue of the Jarník–Besicovitch theorem) With $\Theta$ and $\psi$ as before, what is the Hausdorff dimension of the set $\{\mathbf x\in \mathbb R^d : C_{H_\Theta ,\psi }(\mathbf x) = 0\}$?

### Question 7.3

(Analogue of the Jarník–Schmidt theorem) With $\Theta$ and $\psi$ as before, what is the Hausdorff dimension of the set $\{\mathbf x\in \mathbb R^d : C_{H_\Theta ,\psi }(\mathbf x) > 0\}$? Does this set have large intersections with nice fractals?

## Appendix A: Hardy fields

In this appendix we briefly recall the definition of a Hardy field and its basic properties.

Given $f:(t_0,\infty )\rightarrow \mathbb R$ and $g:(t_1,\infty )\rightarrow \mathbb R$, we write $f\sim g$ if $f(x) = g(x)$ for all sufficiently large *x*.

### Definition A.1

A *Hardy field* is a collection of continuous functions^7^ $\mathcal H$ with the following properties:

(I) For each $f\in \mathcal H$, there exists $t_0\in \mathbb R$ such that $f:(t_0,\infty )\rightarrow \mathbb R$.
(II) Given $f,g\in \mathcal H$, there exist $h_1,h_2,h_3,h_4,h_5\in \mathcal H$ such that $f + g \sim h_1$, $f - g \sim h_2$, $fg \sim h_3$, $f/g \sim h_4$, and $f' \sim h_5$.

The two primary examples of Hardy fields are the field of rational functions and the field of Hardy *L*-functions, described in the introduction. The fact that the collection of Hardy *L*-functions forms a Hardy field was proven by Hardy [3, Theorem 1].

The most important fact about Hardy fields follows almost directly from the definition:

### Lemma A.2

If $f,g\in \mathcal H$ then either

$$\begin{aligned} f(x)\ge g(x) \text { for all} \ x \ \text {sufficiently large} \end{aligned}$$

or

$$\begin{aligned} f(x)\le g(x) \text { for all} \ x\ \text {sufficiently large.} \end{aligned}$$

### Proof

Write $h\sim g - f$ for some $h\in \mathcal H$. Then there exists a function $j\in \mathcal H$ such that $j \sim 1/h$. It follows that $h(x)\ne 0$ for all sufficiently large *x*. Since *h* is continuous, the conclusion follows. $\square$

The following well-known lemma says that in a Hardy field, we can take the derivative of an inequality.

### Lemma A.3

If $f,g\in \mathcal H$, $0\le f(x)\le g(x)$ for all *x* sufficiently large, and $g(x)\rightarrow 0$, then

$$\begin{aligned} |f'(x)| \le |g'(x)| \text { for all} \ x\ \text {sufficiently large}. \end{aligned}$$

### Proof

Write $h \sim g - f$ for some $h\in \mathcal H$; then $0\le f(x),h(x)$ for all *x* sufficiently large, and $f(x),h(x)\rightarrow 0$. It follows that *f* and *h* are eventually decreasing, i.e. $f'(x),h'(x) \le 0$ for all *x* sufficiently large. Rearranging completes the proof. $\square$

One last lemma which we needed in verifying the hypotheses of Lemma 2.4 (cf. Remark 2.5):

### Lemma A.4

If $f\in \mathcal H$ satisfies $Cx \ge f(x) \rightarrow \infty$ for some $C > 0$, then *f* is uniformly continuous and increasing.

### Proof

By Lemma A.3, we have $|f'(x)| \le C$, i.e. $|f'|$ is uniformly bounded. This implies that *f* is uniformly continuous. On the other hand, by Lemma A.3 we have either $f'(x) \ge 0$ for all sufficiently large *x*, or $f'(x) \le 0$ for all sufficiently large *x*. The second case is ruled out since $f(x)\rightarrow \infty$, so *f* is increasing.

## Footnotes

1 Although what we call here the “standard height function” is the most commonly considered height function in the field of Diophantine approximation, a slightly different height function is considered to be standard in other areas of number theory. Namely, if a rational $\mathbf p/q\in \mathbb Q^d$ is in reduced form, then many number theorists, motivated by projective geometry, define the height of $\mathbf p/q$ to be the number $\max (|p_1|,\ldots ,|p_d|,q)$ rather than *q*. The two height functions agree on rationals in the unit cube $[0,1]^d$, as well as agreeing up to a multiplicative error term on bounded subsets of $\mathbb R^d$, so the difference is rarely significant.

2 It has been pointed out to us that there are definitions of the term “height function” according to which $H_{\mathtt{min}}$ is not a height function, since its sublevelsets $\{H_{\mathtt{min}}\le q\}$ ($q\in \mathbb N$) are not discrete (i.e. it does not satisfy the Northcott property on compact sets). However, for our purposes it is not important (except for one place where we must be slightly careful, see Footnote below) whether the sublevelsets of $H_{\mathtt{min}}$ are discrete, and we feel that the role played by $H_{\mathtt{min}}$ in this paper is sufficiently “height-function-like” (in a Diophantine approximation sense) to justify the use of the terminology.

3 There is a subtle distinction here: the existence of infinitely many rational points satisfying a given inequality, versus the existence of a sequence of rational points satisfying the given inequality and tending to the given point $\mathbf x$. This distinction is important in what follows because otherwise the function $H_{\mathtt{min}}$ would behave pathologically, since there exists a bounded region containing infinitely many points $\mathbf r\in \mathbb Q^d$ satisfying $H_{\mathtt{min}}(\mathbf r) = 1$. But we do not want to say that such a sequence is a sequence of “approximations” of a given point $\mathbf x$ unless the sequence actually converges to the point $\mathbf x$ (which could only happen if $\mathbf x$ has an integer coordinate).

4 Technically, there may be more than one optimally Dirichlet function, as shown in [2, Remark 2.11]. However, in this paper we are really only interested in *Hardy L-functions* (which will be defined shortly), and for these functions, there is up to a multiplicative constant at most one optimally Dirichlet function (again see [2, Remark 2.11]).

5 Hardy *L*-functions were defined by Hardy and were originally called *logarithmico-exponential functions*; see [3, §3].

6 Here “best approximations” means “best approximations of the first kind” in the language of [4, p. 24]. Note that if no error term is allowed, then best approximations of the first kind must be *intermediate fractions* (cf. (2.4)), but they are not necessarily convergents.
